# Volatile Organic Compound Monitoring during Extreme Wildfires: Assessing the Potential of Sensors Based on LbL and Sputtering Films

**DOI:** 10.3390/s22176677

**Published:** 2022-09-03

**Authors:** Cátia Magro, Oriana C. Gonçalves, Marcelo Morais, Paulo A. Ribeiro, Susana Sério, Pedro Vieira, Maria Raposo

**Affiliations:** 1 Department of Physics, NOVA School of Science and Technology, NOVA University Lisbon, 2829-516 Almada, Portugal; 2 School for International Training, World Learning Inc., Brattleboro, VT 05302, USA; 3 Centro de Química Estrutural, Institute of Molecular Sciences, Departamento de Química e Bioquímica, Faculdade de Ciências, Universidade de Lisboa, Campo Grande, 1749-016 Lisboa, Portugal; 4 Laboratory of Instrumentation, Biomedical Engineering and Radiation Physics (LIBPhys-UNL), Department of Physics, NOVA School of Science and Technology, NOVA University Lisbon, 2829-516 Almada, Portugal

**Keywords:** extreme wildfires, volatile organic compounds, eucalyptol, α-pinene, electronic nose, impedance spectroscopy, layer-by-layer, sputtering

## Abstract

A new theory suggests that flammable gases generated by heated vegetation, in particular the volatile organic compounds (VOC) common to Mediterranean plants, may, under certain topographic and wind conditions, accumulate in locations where, after the arrival of the ignition source, they rapidly burst into flames as explosions. Hence, there is a need for the development of a system that can monitor the development of these compounds. In this work, a sensor’s array is proposed as a method for monitoring the amount of eucalyptol and α-pinene, the major VOC compounds of the Eucalyptus and Pine trees. The detection of the target compounds was assessed using the impedance spectroscopy response of thin films. Combinations of layers of polyelectrolytes, such as poly(allylamine hydrochloride) (PAH), polyethyleneimine (PEI), poly(sodium 4-sytrenesulfonate) (PSS) graphene oxide (GO), and non/functionalized multiwall nanotubes (MWCNT-COOH or MWCNT), namely, PAH/GO, PEI/PSS, PEI/GO, PAH/MWCNT, PAH/MWCNT-COOH, films, and TiO_2_ and ZnO sputtered films, were deposited onto ceramic supports coated with gold interdigitated electrodes. The results showed that concentrations of the target VOCs, within the range of 68 to 999 ppm_v_, can be easily distinguished by analyzing the impedance spectra, particularly in the case of the ZnO- and PAH/GO-film-based sensors, which showed the best results in the detection of the target compounds. Through principal component analysis (PCA), the best set of features attained for the ZnO and PAH/GO based sensor devices revealed a linear trend of the PCA’s first principal component with the concentration within the range 109 and 807 ppm_v_. Thus, the values of sensitivity to eucalyptol and α-pinene concentrations, which were (2.2 ± 0.3) × 10^−4^ and (5.0 ± 0.7) × 10^−5^ per decade, respectively, as well as resolutions of 118 and 136 ppb_v_, respectively, were identified.

## 1. Introduction

Extreme fire events arise from the combination of extreme weather conditions with land management and even human activities, causing disturbing repercussions at both the ecological and socio-economic levels. Climate change has dramatically increased the occurrence of extreme heat surges and periods of drought in recent years. As a result, ecosystems have become more vulnerable due to fuel accumulation and forest growth, prolonging the duration, size, and intensity of wildfires. In Portugal, the forest area covers 36.1% of the national territory, where several wildfires were recorded over the last decade (2010–2019) that affected approximately 11,000 and 524,000 ha. The most significantly affected tree species were Eucalyptus (*Eucalyptus globulus* Labill.), Maritime pine (*Pinus pinaster* Aiton), Cork oak (*Quercus suber* L.), Holm oak (*Quercus ilex*), Stone pine (*Pinus pinea*), and other oaks (e.g., *Quercus robur* L.). Currently, these species correspond to the most representative species in the Portuguese forests, comprising 25.7, 20.4, 23.3, 11.3, 6.2, and 3.0% of the abundance on the mainland, respectively [1,2,3,4,5,6].

Wildfires can reach frontal speeds of 40 m s^−1^ and temperatures of up to 1500 °C. They are influenced simultaneously by different factors, namely topography, vegetation type, and meteorological parameters, such as air temperature, humidity, precipitation, solar radiation, and wind speed and direction. In particular, forest stands dominated by *E. globulus* Labill. have been shown to be more prone to the incidence of extreme fires due to both the flammability of the species’ semi-separated shell and the emission of various volatile fuels, which can cause long-distance fire outbreaks. Similarly, other plant species have been demonstrated to emit highly reactive volatile substances upon heating, particularly many of the volatile organic compounds (VOC), such as α-pinene. Some studies indicate that the concentration of VOCs is directly proportional to the height of the flame and inversely proportional to the time of fire ignition. In fact, due to their density being more significant than air, it is estimated that, in periods of drought, when the vegetation is more flammable, VOCs tend to accumulate at low altitudes in concentrations close to the lower flammability limit (LFL), approximately of the order of 1% (*v*/*v*) [7]. The fuel gas LFL corresponds to the minimum concentration in the air that is sufficient to promote its ignition [8]. Thus, in the presence of favorable weather conditions and other combustion gases, many accumulated VOCs can cause a rapid and intense ignition, accelerating the propagation of the fire [9,10,11,12,13].

Several international organizations, such as the European Forest Fire Information System (EFFIS) and the Global Wildfire Information System (GWIS), have emerged in recent years, aiming to understand and predict the possibility of wildfires. However, implementing new strategies and measures for the prevention of extreme wildfires remains essential, particularly for determining the various factors that can suddenly change the behavior of fires. Thus, there is a need to create systems that enable the monitoring of the emissions and concentrations of these compounds, making it possible to assess the inherent risk of fire, enhancing the decision support tools, and, therefore, reducing the time between the start of an ignition and the arrival of teams to extinguish the fire [3].

Different studies have been carried out in order to investigate the emission of VOCs emitted from biogenic sources [14,15,16]. However, although the implemented approaches have shown consistent results, their use normally requires qualified personnel and high associated costs [17,18]. These constraints have led to the use of sensor devices as a support for fast and portable monitoring techniques. The electronic noses (e-nose) have been used in several areas, offering the possibility of their use for environmental control and enabling the detection of several VOCs as a simple, fast, portable, and low-cost alternative [19,20]. In an e-nose, different sensors can be used to enable and facilitate the detection of different compounds. This detection is possible due to interactions that occur on the sensor surface when it is in contact with the gas headspace [21,22]. Due to the versatility of e-noses, there has developed an incremental interest in sensor applications aimed towards different purposes. Lieberzeit et al. (2009) analyzed the potential of a molecularly imprinted polymer (MIP) to identify terpenoids (α-pinene, thymol, estragol, linalool, and camphor) emitted by the fresh leaves of herbaceous plants, identifying the selectivity at 40 ppm [23]. Iqbal et al. (2010) [24] reported on the use of a piezoelectric 10 MHz multichannel quartz crystal microbalance coated with six molecularly imprinted polystyrene artificial recognition membranes for the quantification of terpenes emitted from fresh and dried Lamiaceae family species, i.e., rosemary (*Rosmarinus officinalis* L.), basil (*Ocimum basilicum*), and sage (*Salvia officinalis*). The authors were able to achieve optimal e-nose parameters, such as layer heights, linearity within a range of 20–250 ppm_v_, and discrimination between α- and β-pinene. Three years later, Hawaria et al. (2013) [25] demonstrated the successful detection of α-pinene in mango fruit using MIP with an interdigitated electrode as a support. In 2019, Szulczyński et al. proposed a fuzzy logic algorithm for the determination of the odor intensity of the binary mixtures of eight odorants: n-Hexane, cyclohexane, toluene, o-xylene, trimethylamine, triethylamine, α-pinene, and β-pinene. In this research, a prototype of an e-nose equipped with eight gas chemical sensors (one photoionization, two electrochemical, and five metal oxide semiconductor sensors) was used [26]. In 2021, Zhou et al. evaluated the floral volatile profile of six Hedychium accessions using HS–SPME–GC–MS and e-nose technology. The authors were able to provide a reference for the establishment of the rapid detection of Hedychium floral volatile profiles [27]. The described studies demonstrated the wide range of applications and trends in the subject of e-noses, making it clear that the comparison between the different analytical measurement methods is difficult, since each one provides different types of information [28].

Nevertheless, the potential of the e-nose for the detection of VOCs is evident and therefore, with this in mind, the aim of the present work was to develop several thin-film-based sensors using the layer-by-layer and the sputtering techniques to enable the detection of α-pinene and eucalyptol (the main VOCs emitted by *E. globulus* Labill. and *P. pinaster* Aiton trees). Transducing was carried out through impedance spectroscopy, and the data analysis was performed using principal component analysis (PCA).

## 2. Materials and Methods

Standards of (+)-α-pinene (α-pinene; C_10_H_16_; 136.24 g mol^−1^; 97%) and eucalyptol (C_10_H_18_O; 154.25 g mol^−1^; 99%) were purchased from Sigma-Aldrich (St. Louis, MO, USA). Ultra-pure water (resistivity of 20.4 MΩ cm at 24 °C) was obtained using a Direct-Q 3 UV system from Millipore (Bedford, MA, USA). Dimethylformamide (DMF) was purchased from Sigma-Aldrich (St. Louis, MO, USA). The argon (Ar), oxygen (O_2_), and nitrogen (N_2_) gases of ≥99.9% purity were all acquired from Air Liquide (Algés, Portugal).

The ceramic-based sensor devices with deposited gold interdigitated electrodes (IDE) were acquired from Metrohm DropSens (Oviedo, Espanha). The devices’ dimensions were 22.8 mm (length) × 7.6 mm (width) × 1 mm (thickness), and each internal “finger” had a 200 μm width, which also corresponds to the spacing between them.

The thin films with multiple layers, obtained by the LbL technique [29], were prepared with poly(allylamine hydrochloride) (PAH), polyethyleneimine (PEI), poly(sodium 4-sytrenesulfonate) (PSS), and graphene oxide (GO), all purchased from Sigma-Aldrich (St. Louis, MO, USA), while the MWCNT and MWCNT-COOH nanotubes (96% purity, 10–30 μm length) were obtained from Nanografi (Ankara, Turkey). Solutions of each polyelectrolyte (PAH, PEI, PSS, and GO) were prepared with a monomer concentration of 10^−2^ M in ultra-pure water, while for MWCNT and MWCNT-COOH, 1 mg mL^−1^ solutions were prepared by dissolving the nanotube powder in a DMF/ultra-pure water mixture (10:90, *v*/*v*). The LbL coatings were deposited through the adsorption of alternate electrically charged layers of the polyelectrolytes onto the solid/liquid interface on the ceramic support with gold IDE. This was achieved by performing consecutive immersions of the support in the different polyelectrolyte solutions and washing away with water after each immersion to remove any unabsorbed polyelectrolyte molecules and to prevent the solutions’ cross-contamination. For the adsorption of the thin films of MWCNT and MWCNT-COOH, magnetic stirring was used to ensure the avoidance of nanotubes agglomerates. The adsorption time used for each electrolyte layer was 60 s and, after the formation of each bilayer, the layer was dried out using N_2_ gas. The whole process was repeated five times to obtain an array of five bilayered thin films with a positive-charged/negative-charged polyelectrolytes, designated as (PAH/GO)_5_, (PEI/GO)_5_, (PEI/PSS)_5_, (PAH/MWCNT)_5_, and (PAH/MWCNT-COOH)_5_.

The TiO_2_ and ZnO thin films were produced by reactive DC magnetron sputtering using titanium and zinc targets (99.99%, Goodfellow, Cambridge, UK), respectively, as well as argon (99.99%, Air Liquid, Paris, France) and oxygen (99.99%, Gás Piedense gases, Setúbal, Portugal). To achieve a base pressure of 10^−4^–10^−5^ Pa (before introducing the sputtering gas), a turbomolecular pump (Pfeiffer TMH 1001, Pfeiffer Vacuum GmbH, Asslar, Germany) was used. Before the sputter deposition of the films, a movable shutter was placed between the target and the supports. The target was pre-sputtered in an Ar atmosphere for 1 min to remove the target surface oxidation. The target-to-support distance was maintained at 100 mm. For each metal-oxide-based device, the sputtering was performed in both 100% O_2_ and 50:50 O_2_/Ar atmospheres, and the other deposition parameters were maintained as constant, differing for each oxide, as summarized in Table 1.

**Figure 1:**
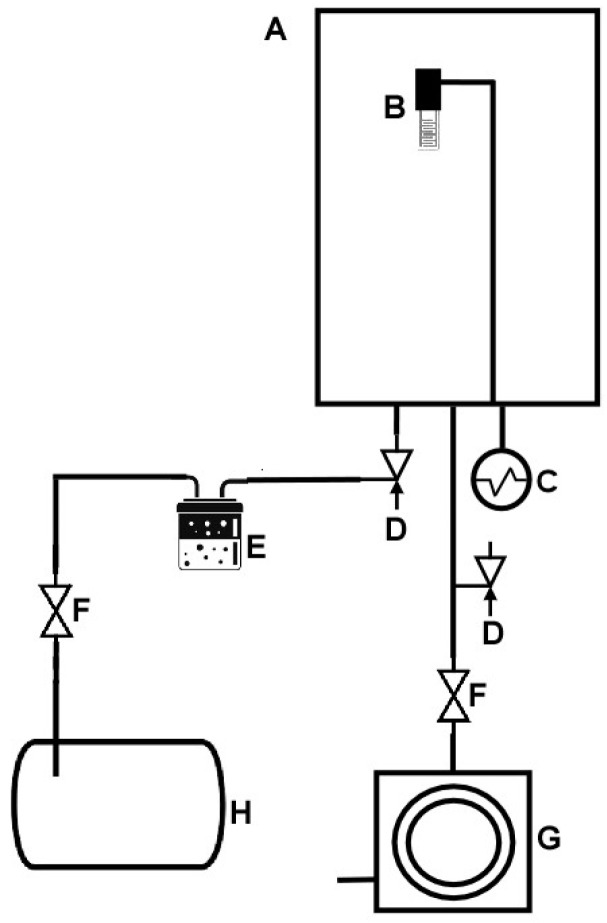
Vacuum system developed for the VOC measurements with the LbL- and sputtered-thin-film-based sensors. (A) vacuum chamber, (B) sensor device, (C) Pirani gauge, (D) inlet needle valve, (E) VOC evaporation flask, (F) gate valve, (G) rotary vacuum pump, (H) compressed reservoir.

A custom-made chamber (Figure 1) was developed to assess the electrical impedance spectra responses of the produced thin films in a controlled atmosphere. In this process, firstly the chamber was evacuated until a pressure of 0.13 Pa was reached, followed by the introduction of a calibrated volume of the selected VOC standard solution into a round-bottomed flask. After closing the system, the sample was vaporized and purged with compressed synthetic air ALPHAGAZ^TM^ 1 AR (Air Liquide, Algés, Portugal) in the measurement chamber containing the nanosensors until a pressure of 1 atm was reached. The electrical response of the sensors to the VOC was then assessed by measuring the impedance spectra with a Solartron 1260 Impedance/Gain-Phase Analyzer coupled with a 1296A Dielectric Interface (Solartron Analytical, AMETEK scientific instruments, Berwyn, PA, USA) and processed with the SMaRT Impedance Measurement Software (v. 3.3.1, AMETEK scientific instruments, Berwyn, PA, USA). The implemented VOC concentrations were within the range between 68 and 990 ppm_v_, whereas the impedance was measured in the frequency range of 1 to 10^6^ Hz, while applying an AC voltage of 25 mV. The implemented concentrations were calculated using the perfect gas equation (P_vapor_ × V = *n*_molecule_ × R × T). Considering the vapor pressure (P_vapor_) value of each compound (253.2 Pa for eucalyptol and 633.3 Pa for α-pinene) and T = 298 K, it was possible to obtain the molecule number of the moles (*n*_molecule_).

The VOC contents inside the measuring chamber were estimated based on the VOC solution volume initially placed in the round-bottomed flask. For the repeatability assessment, the assays were performed in triplicate for each sensor, while for the reproducibility, two different sensors of each thin film were tested.

The principal component analysis (PCA) was carried out considering the normalized impedance spectroscopy data (*Z*-score normalization: $z=\frac{x-\mu }{\sigma }$, with *μ* and *σ* as the mean value and standard deviation of the samples, respectively). This allowed us to reduce the size of data while obtaining a new area of orthogonal components, whose different concentration patterns could be examined and explained. Moreover, an array of the nanostructured sensors constituted by all produced thin-films were assessed as an e-nose for both α-pinene and eucalyptol detection in air. All assays were performed in duplicate.

The sensitivity and resolution were obtained using the following equation:

(1) $$\Delta logC=logC-logC_{s}$$

where $\Delta logC=\frac{error}{sensitivity}$ and, therefore, the resolution is equal to $C-C_{s}$. The sensitivity corresponds to the slope of the linear function that adequately fits the plotted data. The resolution corresponds to the lowest concentration that can be detected (the limit of detection) and may be found near the smallest concentration of the implicit linear range (*C_S_*).

## 3. Results and Discussion

### 3.1. Assessment of the Sensor Responses to VOC in Air: Impedance Spectroscopy

**Figure 2:**
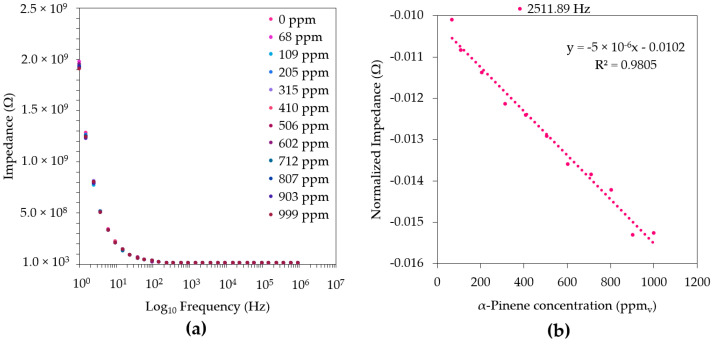
Impedance spectra of a (PAH/GO)_5_-film-based sensor (**a**,**b**) with its normalized impedance (Ω) spectra at a fixed frequency (2511.89 Hz) for detecting different α-pinene concentrations in air. The average and standard deviations of the respective impedance data of the duplicates were used for the normalization (Supplementary Materials, Table S9).

The sensorial response of each thin film was assessed through the impedance spectra measurements at different gas-phase concentrations of α-pinene and eucalyptol in the frequency range of 1 to 10^6^ Hz. The obtention of the characteristic footprints for each sensor device revealed that, for all the LbL thin-films, no evident differentiation between the compounds’ concentrations was observed within the frequency range studied (Supplementary Materials, Figures S1a–d and S2a–d). However, at a fixed frequency of 3.98 Hz, the (PAH/GO)_5_-based sensor exhibited the greatest efficiency for α-pinene quantification, revealing a linear tendency that was inversely proportional to the compound’s concentration, with a correlation coefficient (*r*^2^) of 0.9805, as shown in Figure 2. Moreover, using the same frequency, this thin film also showed great repeatability in this VOC assessment (residual standard deviation, RSD ≤ 1.9%).

The thin films produced using the LbL technique were highly morphologically organized in a layered form, as usual, as this approach enables the consistent management of the films’ thickness and structure, which leads to the selective adsorption of molecules in specific regions on its surface. The film stability is caused by electrostatic interactions established by the different polyelectrolytes [30]. In the case of both the LbL thin films with the same negative charged polyelectrolyte, i.e., (PEI/GO)_5_ (Supplementary Materials, Figures S1b and S2a) and (PAH/GO)_5_, PEI’s monomeric structure was constituted by a secondary amine group, while PAH molecules had a primary amine. Since the nitrogen atom in a primary amine is bound to two hydrogen atoms instead of one, it is a less stereo-chemically hindered group, which leads to a stronger nucleophilicity compared to a secondary one [31]. Hence, PAH is more likely to react with the highly electrophilic groups present on the GO surface, such as epoxy groups, thus forming a stronger and better arranged polyelectrolyte layer. Thus, the formed PAH/GO bilayer should be more organized and stable than the PEI/GO one, enabling a better detection of the molecules, which might explain the results obtained for α-pinene (Supplementary Material Figure S2a). However, both previously mentioned thin films showed a lack of efficiency in the eucalyptol measurements, which may be related to the strong interactions that occur between this molecule and the GO surface. As the ideal LbL thin film should repel the target compound in order to avoid both adsorption and desorption phenomena [30], it is important to contemplate the different interactions that may take place between the molecules and the sensor’s surface. The retention of diverse organic molecules on nanostructured materials can be effected by both chemisorption and physisorption processes. In chemisorption, a chemical reaction takes place between the molecule and the material’s surface, while in physisorption, the retention occurs through interactions that do not involve direct covalent bonding, such as van der Waals forces, hydrogen bonds, and even electrostatic, reverse-phase, and π−π interactions. Overall, chemisorption leads to a stronger and even irreversible retention of the molecules, followed by electrostatic interactions and hydrogen bonds. Thus, the presence of this type of strong interactions is not beneficial for a sensor’s coating, as they can lead to an irreversible adsorption of the molecule and, consequently, a poorer detection [32,33]. Therefore, the double carbon–carbon bond in α-pinene’s chemical structure might allow the molecule to be retained by the GO surface merely through reverse-phase, π−π, and van der Waals interactions. In contrast, the structure of eucalyptol has an oxygen atom, meaning that both electrostatic interactions and hydrogen bonds also play a role in the molecule’s retention by the GO layer [34]. As these types of interactions are stronger than the ones established with the α-pinene molecule, eucalyptol’s adsorption on the GO-based thin films might be stronger than that of α-pinene, which results in a less efficient desorption step and, therefore, a lower sensor detection performance.

**Figure 3:**
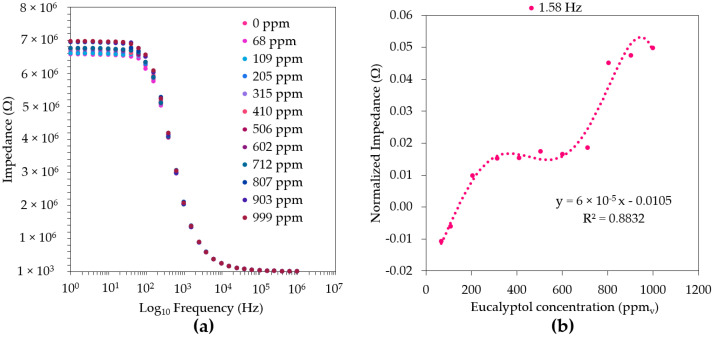
Impedance spectra of the TiO_2_ (100% O_2_)-film-based sensor (**a**) and its normalized impedance (Ω) spectra at a fixed frequency (1.58 Hz) for different eucalyptol concentrations in air (**b**). The dotted line is only a guideline. The average and standard deviations of the respective impedance data of the duplicates were used for the normalization (Supplementary Materials, Table S8).

As the mechanisms of the interactions between the molecules in the gas-phase and a material involve both physical and chemical sorption processes, the sensors’ sensitivity can also be highly affected by the thin films’ surface imperfections. According to previous studies [35], LbL films, such as (PAH/GO)_5_ and (PEI/GO)_5_, exhibit highly structured and smooth surfaces, which are different from the (PAH/MWCNT)_5_ and (PAH/MWCNT)_5_ coatings and show a more irregular outer layer [36]. This may be a result of the low scattering tendency of the MWCNTs structures [36], which leads to cluster formation through the agglomeration of these structures. Furthermore, the surface’s morphology is dependent on the deposition technique used. In the case of the LbL technique, the thin films tend to exhibit a smoother and highly organized surface, while the sputtering methodology leads to the formation of rougher surfaces and agglomerates of different shapes [35]. Moreover, according to Magro et al. (2022) [37], an oxygen-rich atmosphere leads to the production of a smoother surface, especially in the case of the ZnO-based films. Similarly, as with the LbL thin-films, a smoother surface can lead to better interactions between the analytes and the thin film surface; thus, it can be expected that both TiO_2_- and ZnO-coated thin films produced and previously studied in other works [35,37] would show a better performance in the determination of the studied compounds. As a result, the electrical footprint of the sputtered thin films obtained in a 50% O_2_/50% Ar atmosphere demonstrated an imprecise distinction between the concentrations of both α-pinene and eucalyptol. Thus, the TiO_2_ (100% O_2_) showed a good differentiation of the diverse eucalyptol concentrations between 1 and 100 Hz (Figure 3a), showing the best fitting efficiency and repeatability (RSD ≤ 0.006%) in its determination at a frequency of 1.58 Hz (Figure 3b).

**Figure 4:**
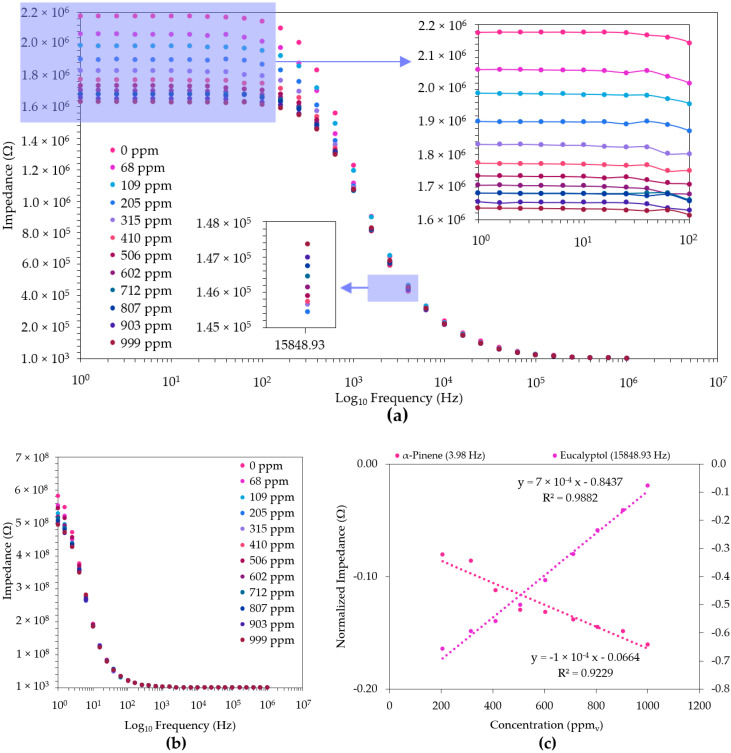
Impedance spectra of the ZnO (100% O_2_)-based film sensor obtained for eucalyptol (**a**) and α-pinene (**b**), and normalized impedance (Ω) spectra at a fixed frequencies (eucalyptol, 15,848.93 Hz; α-pinene, 3.98 Hz, (**c**) for the measurement of different concentrations in air. The average and standard deviations of the respective impedance data of the duplicates were used for the normalization (Supplementary Materials, Tables S6 and S14).

Nevertheless, amongst all the produced thin films, the ZnO-based sensors obtained in a 100% O_2_ atmosphere were the only ones that showed a significant ability for the discrimination of the diverse concentrations of both eucalyptol and α-pinene, as can be observed in Figure 4a,b, respectively.

Similarly, at the fixed frequencies of 15,848.93 Hz (eucalyptol) and 3.98 Hz (α-pinene), these thin films exhibited a high linearity (*r* ≥ 0.92) in the quantification of eucalyptol and α-pinene, respectively, as can be observed in Figure 4c. Furthermore, as expected according to the discrimination observed in Figure 4b, in the lower frequencies, the thin films showed good repeatability (RSD ≤ 14.4%) for the α-pinene assessment at 3.98 Hz. However, this was not verified for the eucalyptol, where a greater repeatability (RSD ≤ 13.9%) was obtained at higher frequencies (e.g., 15,848.93 Hz), where the capacity for concentration discrimination was not as strong as that seen in the frequency range of 1 to 100 Hz (Figure 4a).

### 3.2. Principal Component Analysis

**Figure 5:**
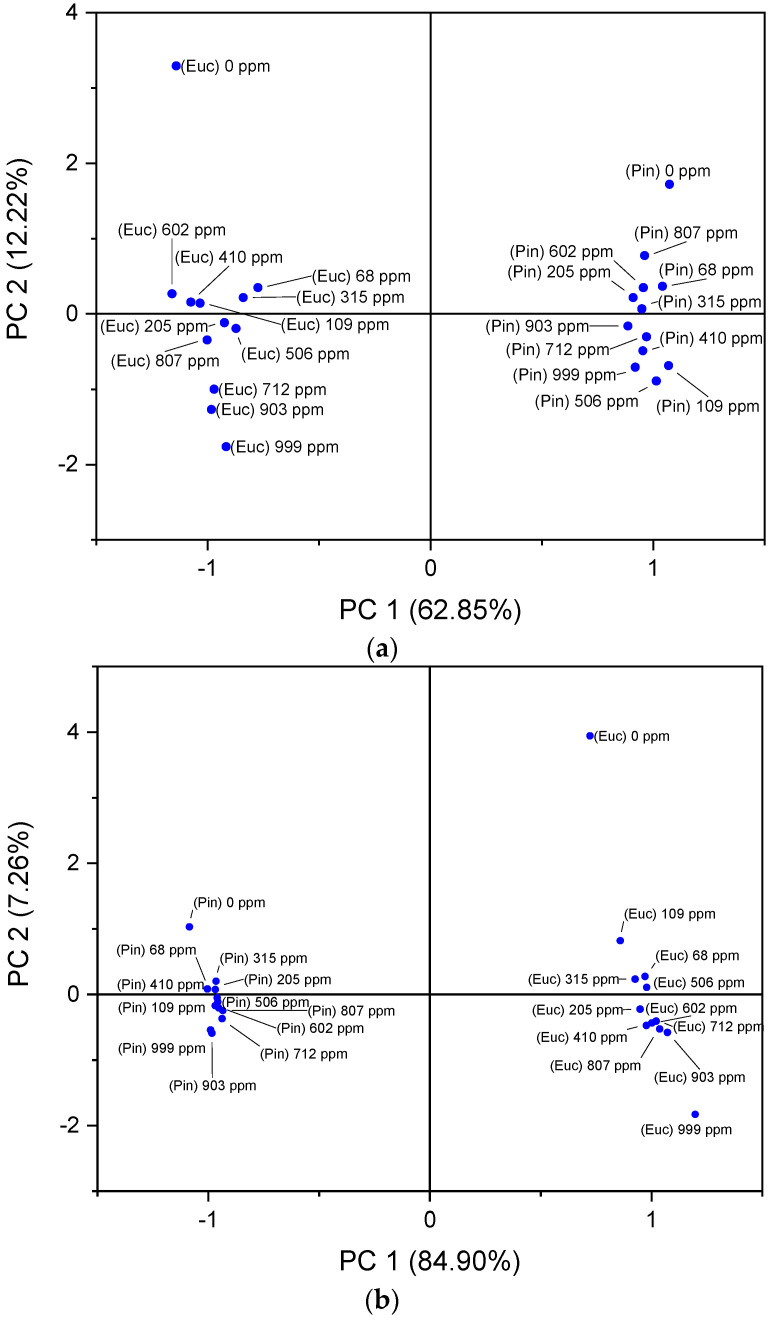
(**a**) PCA score plot for the electronic nose concept, using the array of PAH/GO, PEI/PSS, PEI/GO, PAH/MWCNT, PAH/MWCNT-COOH, TiO_2_ and ZnO sensors for the detection of eucalyptol and α-pinene in the concentration range of 68 to 999 ppm_v_. (**b**) PCA score plot for the electronic nose concept, using the array of ZnO (100% O_2_)- and (PAH/GO)_5_-film-based sensors for the detection of eucalyptol and α-pinene, respectively, in the concentration range of 68 to 999 ppm_v_. A concentration value of 0 ppm_v_ of both VOCs were used as the blank samples.

Following the analysis of the electrical responses and capabilities of the nanostructured thin-film-based sensors, the electronic nose concept was evaluated. Using the impedance, capacitance, resistance, reactance, and loss tangent data, the array of sensors were established as an e-nose. Figure 5a,b shows the array composed of all the thin film sensors produced in the present study and the best performing sensors for both target compounds, respectively.

Overall, the two sensor arrays showed an ability to distinguish eucalyptol and α-pinene, and within the different compound clusters, it was able to discriminate between blank samples and air samples containing different concentrations of both volatiles. The e-nose, accounting for the data of all sensors (Figure 5a), explained 75.1% of the total variance, in contrast with 92.2% of the total variance when the best performance sensors were used as the detection device. Considering Figure 5a, although the PCA plot distinguishes the different eucalyptol and α-pinene concentrations, no trends or patterns were observed. On the contrary, the PCA plot in Figure 5b clearly distinguishes the different concentrations across the main axis, PC 1, where a pattern and a decreasing trend with the increasing concentrations throughout PC 1 are also observed.

**Figure 6:**
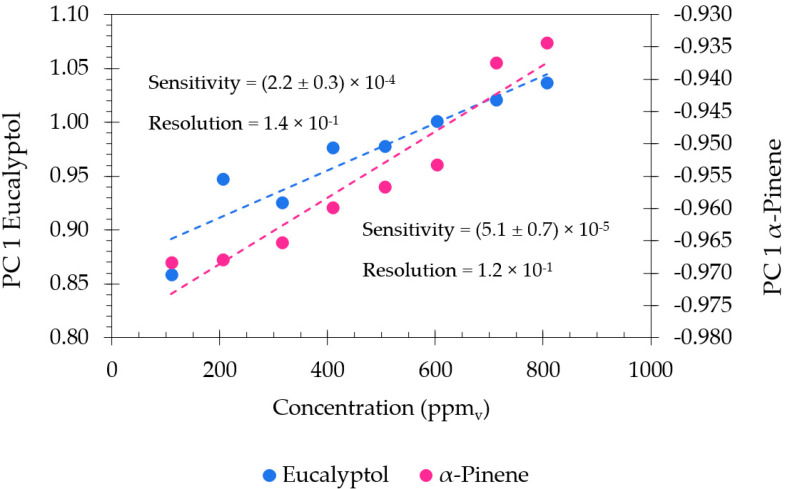
Eucalyptol and α-pinene sensitivity and resolution values achieved using a sensor array of ZnO (100% O_2_) and (PAH/GO)_5_. PC1 factor scores as a function of the eucalyptol and α-pinene concentrations, ranging from 109 to 807 ppm_v_.

Furthermore, the factor scores of PC 1 obtained in Figure 5b were plotted as a function of the eucalyptol and α-pinene concentrations in order to identify the sensitivity and the resolution of the present device (Figure 6).

The best set of sensors, ZnO and PAH/GO for eucalyptol and α-pinene, respectively, through the PCA first principal component showed a linear trend with the concentration in the range between 109 and 807 ppm_v_. Thus, the values of sensitivity to the eucalyptol and α-pinene concentrations, (2.2 ± 0.3) × 10^−4^ and (5.1 ± 0.7) × 10^−5^ per decade, respectively, were achieved. The resolutions were in the range of 118 and 136 ppb_v_, respectively.

## 4. Conclusions

An e-nose based on an array of sensors was developed to monitor the concentration of eucalyptol and α-pinene, highly reactive volatile substances upon heating, which are produced by Eucalyptus and Pine trees, respectively, and which strongly contribute to wildfires. The detection of these target compounds was achieved by preparing an array of layer-by-layer thin films, including (PAH/GO)_5_, (PEI/PSS)_5_, (PEI/GO)_5_, (PAH/MWCNT)_5_, (PAH/MWCNT-COOH)_5_, and the sputtered thin films TiO_2_ and ZnO, which were deposited onto ceramic substrates with gold interdigitated electrodes. The studied concentration range of the target compounds ranged from 0 to 999 ppm_v_. The detection was based on impedance spectroscopy, providing the experimental data examined by the PCA method. Our results clearly demonstrated that:

1. The electrical response of the sensors was influenced by the different concentrations of eucalyptol and α-pinene;
2. Sensors produced with thin films of metallic oxides showed the best results;
3. The detection of different molecules was influenced by the surface morphology of the thin films that were developed;
4. The use of PAH as a positive polyelectrolyte may positively influence the adsorption of α-pinene;
5. The e-nose system enabled a clear separation of the two target compounds.

These conclusions further show that the developed e-nose might have the potential to predict the imminence of a wildfire by detecting the increases in eucalyptol and α-pinene concentrations in a forest.

## Figures and Tables

**Table 1:** Sputtering parameters used for the deposition of the ZnO and TiO_2_ thin films onto ceramic-based supports with gold IDE.

Thin Film *	O_2_ (%)	Ar (%)	Power (W)	Voltage (V)	Electric Current (A)
ZnO	100	0	300	287	1.04
ZnO	50	50	300	380	0.80
TiO_2_	100	0	1000	326	3.14
TiO_2_	50	50	1000	341	3.19

* Work pressure = 0.8 Pa; deposition time = 5 min.

## Data Availability

Not applicable.

## Supplementary Materials

The following supporting information can be downloaded at: https://www.mdpi.com/article/10.3390/s22176677/s1, Figure S1. Impedance spectra of the (PAH/GO)_5_ (a), (PEI/GO)_5_ (b), (PAH/MWCNT)_5_ (c), (PAH/MWCNT-COOH)_5_ (d), ZnO prepared with 50% O_2_ (e), and TiO_2_ prepared with 100% O_2_ (f) thin films for different eucalyptol concentrations in air. The average and standard deviations of the respective impedance data of the duplicates were used for the normalization; Figure S2. Impedance spectra of the (PEI/GO)_5_ (a), (PAH/MWCNT)_5_ (b), (PAH/MWCNT-COOH)_5_ (c), ZnO prepared with 50% O_2_ (d), TiO_2_ prepared with 100% O_2_ (e), and TiO_2_ prepared with 50% O_2_ (f) thin films for different α-pinene concentrations in air. The average and standard deviations of the respective impedance data of the duplicates were used for the normalization; Table S1. Standard deviation of the impedance measurements of eucalyptol obtained with the (PAH/GO)_5_-thin-film-based sensor at different frequencies; Table S2. Standard deviation of the impedance measurements of eucalyptol obtained with the (PEI/GO)_5_-thin-film-based sensor at different frequencies; Table S3. Standard deviation of the impedance measurements of eucalyptol obtained with the (PAH/MWCNT)_5_-thin-film-based sensor at different frequencies; Table S4. Standard deviation of the impedance measurements of eucalyptol obtained with the (PAH/MWCNT-COOH)_5_-thin-film-based sensor at different frequencies; Table S5. Standard deviation of the impedance measurements of eucalyptol obtained with the ZnO (50% O_2_)-thin-film-based sensor at different frequencies; Table S6. Standard deviation of the impedance measurements of eucalyptol obtained with the ZnO (100% O_2_)-thin-film-based sensor at different frequencies; Table S7. Standard deviation of the impedance measurements of eucalyptol obtained with the TiO_2_ (50% O_2_)-thin-film-based sensor at different frequencies; Table S8. Standard deviation of the impedance measurements of eucalyptol obtained with the TiO_2_ (100% O_2_)-thin-film-based sensor at different frequencies; Table S9. Standard deviation of the impedance measurements of α-pinene obtained with the (PAH/GO)_5_-thin-film-based sensor at different frequencies; Table S10. Standard deviation of the impedance measurements of α-pinene obtained with the (PEI/GO)_5_-thin-film-based sensor at different frequencies; Table S11. Standard deviation of the impedance measurements of α-pinene obtained with the (PAH/MWCNT)_5_-thin-film-based sensor at different frequencies; Table S12. Standard deviation of the impedance measurements of α-pinene obtained with the (PAH/MWCNT-COOH)_5_-thin-film-based sensor at different frequencies; Table S13. Standard deviation of the impedance measurements of α-pinene obtained with the ZnO (50% O_2_)-thin-film-based sensor at different frequencies; Table S14. Standard deviation of the impedance measurements of α-pinene obtained with the ZnO (100% O_2_)-thin-film-based sensor at different frequencies; Table S15. Standard deviation of the impedance measurements of α-pinene obtained with the TiO_2_ (50% O_2_)-thin-film-based sensor at different frequencies; Table S16. Standard deviation of the impedance measurements of α-pinene obtained with the TiO_2_ (100% O_2_)-thin-film-based sensor at different frequencies.
