# Supplementary material for: Volatile Organic Compound Monitoring during Extreme Wildfires: Assessing the Potential of Sensors Based on LbL and Sputtering Films
Source: Sensors (Basel). 2022 Sep 3;22(17):6677. doi: 10.3390/s22176677 (PMC9460900; doi:10.3390/s22176677)
Supplement: Supplementary file 1 [file sensors-22-06677-s001.zip › sensors-1888861-supplementary.pdf]

Supplementary Material

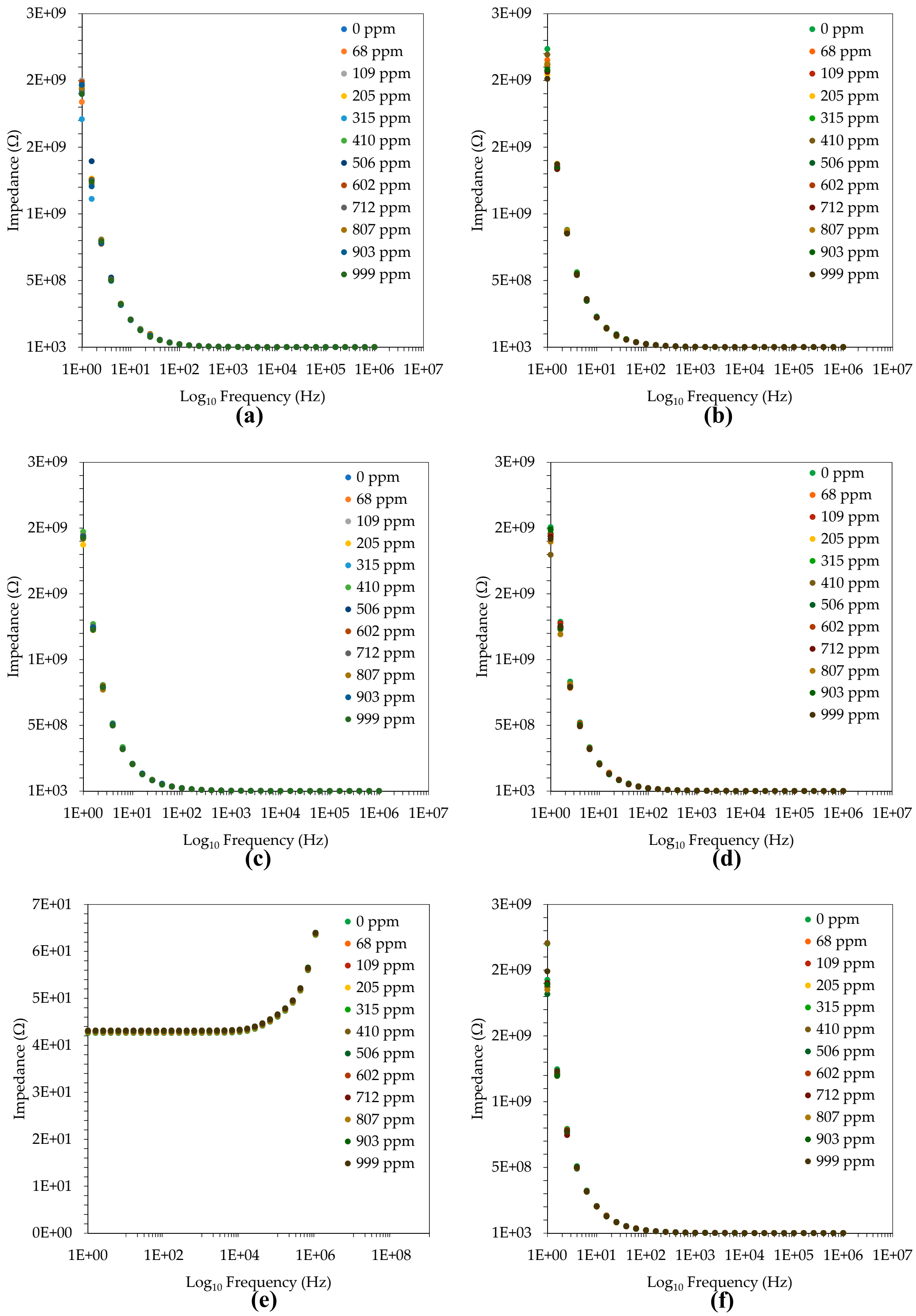

Figure S1. Impedance spectra of the $(PAH/GO)_5$ (a), $(PEI/GO)_5$ (b), $(PAH/MWCNT)_5$ (c), $(PAH/MWCNT\text{-}COOH)_5$ (d), ZnO prepared with 50% O_2 (e), and TiO_2 prepared with 100% O_2 (f) thin-films for different eucalyptol concentrations in air. The average and standard deviations of the respective impedance data of triplicates were used for the normalization.

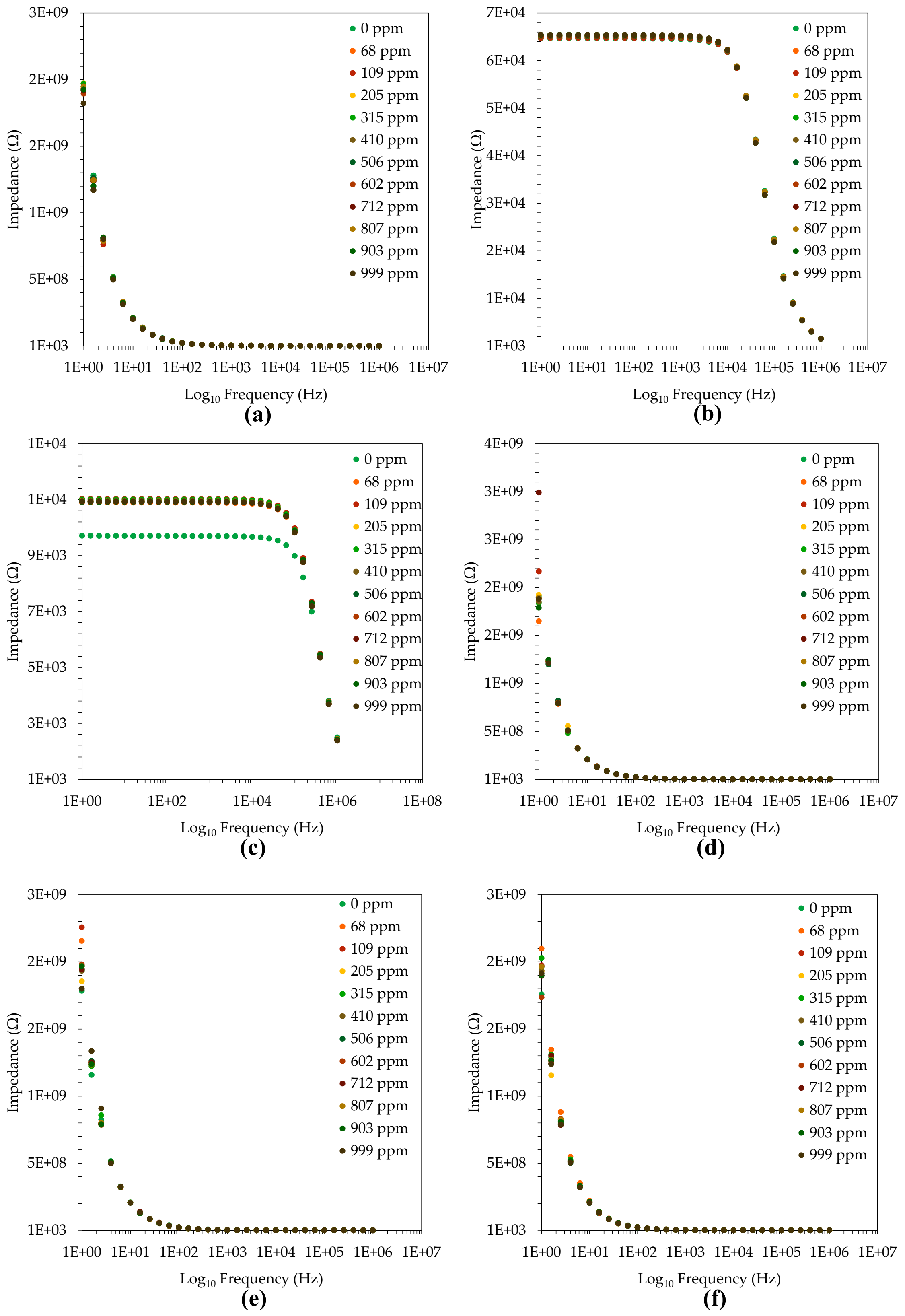

Figure S2. Impedance spectra of the (PEI/GO)$_5$ (a), (PAH/MWCNT)$_5$ (b), (PAH/MWCNT-COOH)$_5$ (d), ZnO prepared with 50% O_2 (e), TiO_2 prepared with 100% O_2 (e), and TiO_2 prepared with 50% O_2 (f) thin-films for different α-pinene concentrations in air. The average and standard deviations of the respective impedance data of triplicates were used for the normalization.

Table S1. Standard deviation of the impedance measurements of eucalyptol obtained with the $(PAH/GO)_5$-thin-film-based sensor at different frequencies.

Log10 Frequency (Hz)	Standard deviation of impedance (Ω)											
	0 ppm	68 ppm	109 ppm	205 ppm	315 ppm	410 ppm	506 ppm	602 ppm	712 ppm	807 ppm	903 ppm	999 ppm
1.0E+06	1.8E+01	2.8E+01	1.3E+01	1.9E+01	3.7E+01	4.5E+01	4.6E+01	3.9E+01	4.1E+01	3.9E+01	4.3E+01	4.5E+01
6.3E+05	2.6E+01	3.3E+01	2.6E+01	2.8E+01	7.1E+01	7.1E+01	7.2E+01	6.9E+01	7.0E+01	6.8E+01	7.1E+01	7.1E+01
4.0E+05	4.0E+01	4.7E+01	4.2E+01	4.3E+01	1.2E+02	1.1E+02	1.2E+02	1.1E+02	1.1E+02	1.1E+02	1.1E+02	1.1E+02
2.5E+05	6.1E+01	7.1E+01	6.7E+01	6.7E+01	1.9E+02	1.8E+02	1.8E+02	1.8E+02	1.8E+02	1.8E+02	1.8E+02	1.8E+02
1.6E+05	9.5E+01	1.1E+02	1.0E+02	1.0E+02	3.0E+02	2.9E+02	2.9E+02	2.9E+02	2.9E+02	2.8E+02	2.9E+02	2.9E+02
1.0E+05	1.6E+02	1.8E+02	1.7E+02	1.7E+02	4.9E+02	4.7E+02	4.7E+02	4.6E+02	4.7E+02	4.6E+02	4.7E+02	4.7E+02
6.3E+04	2.4E+02	2.8E+02	2.7E+02	2.6E+02	7.7E+02	7.4E+02	7.4E+02	7.3E+02	7.4E+02	7.2E+02	7.4E+02	7.4E+02
4.0E+04	3.8E+02	4.4E+02	4.1E+02	4.1E+02	1.2E+03	1.2E+03	1.2E+03	1.2E+03	1.2E+03	1.1E+03	1.2E+03	1.2E+03
2.5E+04	5.8E+02	6.8E+02	6.5E+02	6.4E+02	1.9E+03	1.8E+03	1.8E+03	1.8E+03	1.8E+03	1.8E+03	1.8E+03	1.9E+03
1.6E+04	8.8E+02	1.0E+03	9.9E+02	9.8E+02	3.0E+03	2.9E+03	2.9E+03	2.8E+03	2.9E+03	2.8E+03	2.9E+03	2.9E+03
1.0E+04	1.4E+03	1.6E+03	1.6E+03	1.5E+03	4.8E+03	4.5E+03	4.6E+03	4.5E+03	4.5E+03	4.4E+03	4.6E+03	4.6E+03
6.3E+03	2.1E+03	2.5E+03	2.4E+03	2.4E+03	7.4E+03	7.1E+03	7.2E+03	7.1E+03	7.1E+03	6.9E+03	7.2E+03	7.2E+03
4.0E+03	3.2E+03	4.1E+03	3.6E+03	3.6E+03	1.2E+04	1.1E+04	1.1E+04	1.1E+04	1.1E+04	1.1E+04	1.1E+04	1.1E+04
2.5E+03	4.8E+03	5.9E+03	5.6E+03	5.6E+03	1.8E+04	1.7E+04	1.7E+04	1.7E+04	1.7E+04	1.7E+04	1.7E+04	1.7E+04
1.6E+03	7.1E+03	8.8E+03	8.4E+03	8.4E+03	2.7E+04	2.6E+04	2.6E+04	2.6E+04	2.6E+04	2.6E+04	2.7E+04	2.7E+04
1.0E+03	1.1E+04	1.5E+04	1.1E+04	1.5E+04	4.0E+04	3.9E+04	3.9E+04	3.7E+04	3.6E+04	4.0E+04	4.0E+04	4.0E+04
6.3E+02	2.3E+04	2.2E+04	1.9E+04	2.3E+04	5.6E+04	5.2E+04	5.0E+04	5.3E+04	5.0E+04	6.1E+04	5.8E+04	5.3E+04
4.0E+02	1.9E+04	5.1E+04	3.3E+04	5.2E+04	7.5E+04	7.7E+04	5.8E+04	7.8E+04	7.1E+04	7.5E+04	8.6E+04	7.3E+04
2.5E+02	1.6E+04	8.6E+04	1.9E+04	1.2E+05	5.2E+04	1.1E+05	5.7E+04	3.9E+04	5.9E+04	7.3E+04	1.2E+05	1.7E+05
1.6E+02	2.1E+04	7.8E+04	5.9E+04	7.5E+04	8.8E+04	1.4E+05	1.3E+05	1.0E+05	8.7E+04	8.1E+04	8.9E+04	1.5E+05
1.0E+02	2.8E+05	4.5E+05	7.8E+05	3.0E+05	4.2E+04	6.9E+05	1.3E+05	1.7E+05	5.3E+05	4.2E+05	2.1E+05	3.3E+05
6.3E+01	5.0E+05	1.4E+06	1.9E+06	1.5E+06	1.5E+06	1.8E+05	4.7E+05	8.8E+05	1.5E+05	1.7E+05	8.0E+05	7.8E+05
4.0E+01	3.1E+05	1.9E+06	4.3E+06	1.6E+06	9.5E+04	2.8E+06	1.9E+05	1.2E+06	1.3E+05	3.7E+06	1.4E+06	1.3E+06
2.5E+01	1.1E+06	2.6E+06	2.2E+05	1.6E+06	1.2E+06	9.9E+05	9.2E+04	2.5E+06	1.1E+06	2.0E+06	1.9E+05	1.2E+04
1.6E+01	1.1E+06	4.5E+06	6.2E+06	2.6E+05	3.9E+06	3.4E+06	2.0E+05	3.9E+06	5.1E+06	6.2E+06	5.3E+06	8.6E+05
1.0E+01	6.2E+05	1.1E+06	8.7E+05	1.2E+06	2.3E+06	1.3E+06	6.8E+05	1.2E+06	8.7E+05	7.9E+05	1.0E+06	7.6E+05
6.3E+00	5.0E+05	3.8E+06	6.9E+05	1.3E+06	5.5E+06	6.3E+05	2.8E+06	2.9E+06	3.2E+06	3.1E+06	2.7E+05	7.5E+05
4.0E+00	5.8E+06	1.8E+06	1.5E+06	4.4E+06	1.0E+07	2.0E+05	4.2E+06	1.2E+05	2.0E+06	3.8E+06	4.5E+06	9.3E+05
2.5E+00	2.9E+06	4.3E+06	1.4E+07	2.2E+06	1.8E+06	2.7E+06	3.9E+06	3.7E+06	9.4E+06	3.9E+06	2.8E+04	9.4E+06
1.6E+00	1.1E+07	4.0E+06	2.6E+06	1.3E+06	5.1E+06	2.3E+06	4.7E+06	4.8E+06	8.5E+06	3.5E+05	1.2E+07	1.9E+07
1.0E+00	2.9E+08	5.4E+07	8.4E+06	1.0E+07	2.0E+07	2.9E+05	3.0E+06	3.0E+07	5.5E+06	2.1E+06	8.4E+06	1.6E+06

Table S2. Standard deviation of the impedance measurements of eucalyptol obtained with the $(PEI/GO)_5$-thin-film-based sensor at different frequencies.

Log10 Frequency (Hz)	Standard deviation of impedance (Ω)											
	0 ppm	68 ppm	109 ppm	205 ppm	315 ppm	410 ppm	506 ppm	602 ppm	712 ppm	807 ppm	903 ppm	999 ppm
1.0E+06	2,0E+02	2,0E+02	2,1E+02	2,0E+02	2,1E+02	2,0E+02	2,1E+02	2,0E+02	2,0E+02	2,0E+02	1,9E+02	2,3E+02
6.3E+05	3,4E+02	3,4E+02	3,4E+02	3,3E+02	3,4E+02	3,2E+02	3,3E+02	3,3E+02	3,2E+02	3,3E+02	3,2E+02	3,9E+02
4.0E+05	3,9E+02	5,4E+02	5,4E+02	5,3E+02	5,4E+02	5,2E+02	5,4E+02	5,2E+02	5,2E+02	5,2E+02	5,1E+02	6,2E+02
2.5E+05	6,1E+02	8,6E+02	8,6E+02	8,4E+02	8,4E+02	8,3E+02	8,5E+02	8,2E+02	8,2E+02	8,3E+02	8,1E+02	9,9E+02
1.6E+05	9,7E+02	1,4E+03	1,4E+03	1,3E+03	1,3E+03	1,3E+03	1,3E+03	1,3E+03	1,3E+03	1,3E+03	1,3E+03	1,6E+03
1.0E+05	1,5E+03	2,2E+03	2,1E+03	2,1E+03	2,1E+03	2,1E+03	2,1E+03	2,1E+03	2,1E+03	2,1E+03	2,0E+03	2,5E+03
6.3E+04	2,4E+03	3,4E+03	3,4E+03	3,3E+03	3,3E+03	3,2E+03	3,2E+03	3,2E+03	3,2E+03	3,2E+03	3,2E+03	3,9E+03
4.0E+04	3,8E+03	5,4E+03	5,3E+03	5,2E+03	5,1E+03	5,1E+03	5,1E+03	5,1E+03	5,1E+03	5,1E+03	5,0E+03	6,1E+03
2.5E+04	6,0E+03	8,4E+03	8,3E+03	8,1E+03	8,0E+03	8,0E+03	8,0E+03	7,9E+03	7,9E+03	7,9E+03	7,8E+03	9,5E+03
1.6E+04	9,4E+03	1,3E+04	1,3E+04	1,3E+04	1,3E+04	1,2E+04	1,3E+04	1,2E+04	1,2E+04	1,2E+04	1,2E+04	1,5E+04
1.0E+04	1,5E+04	2,1E+04	2,0E+04	2,0E+04	2,0E+04	2,0E+04	2,0E+04	1,9E+04	1,9E+04	1,9E+04	1,9E+04	2,3E+04
6.3E+03	2,3E+04	3,2E+04	3,2E+04	3,1E+04	3,1E+04	3,1E+04	3,1E+04	3,0E+04	3,0E+04	3,0E+04	3,0E+04	3,5E+04
4.0E+03	3,6E+04	5,1E+04	5,0E+04	4,9E+04	4,8E+04	4,8E+04	4,8E+04	4,7E+04	4,7E+04	4,7E+04	4,7E+04	5,4E+04
2.5E+03	5,7E+04	7,9E+04	7,8E+04	7,6E+04	7,5E+04	7,5E+04	7,4E+04	7,4E+04	7,4E+04	7,4E+04	7,3E+04	8,2E+04
1.6E+03	8,9E+04	1,2E+05	1,2E+05	1,2E+05	1,2E+05	1,2E+05	1,2E+05	1,2E+05	1,2E+05	1,2E+05	1,1E+05	1,3E+05

1.0E+03	1,4E+05	1,9E+05	1,9E+05	1,9E+05	1,8E+05	1,8E+05	1,8E+05	1,8E+05	1,8E+05	1,8E+05	1,8E+05	1,9E+05
6.3E+02	2,2E+05	3,0E+05	3,0E+05	2,9E+05	2,8E+05	2,9E+05	2,8E+05	2,7E+05	2,9E+05	2,8E+05	2,8E+05	3,0E+05
4.0E+02	3,3E+05	4,6E+05	4,8E+05	4,6E+05	4,4E+05	4,3E+05	4,4E+05	4,5E+05	4,6E+05	4,3E+05	4,4E+05	4,8E+05
2.5E+02	4,9E+05	8,3E+05	7,1E+05	6,6E+05	7,2E+05	6,7E+05	6,6E+05	5,9E+05	6,8E+05	6,8E+05	6,3E+05	7,8E+05
1.6E+02	8,3E+05	1,1E+06	1,1E+06	1,1E+06	1,1E+06	1,1E+06	1,1E+06	1,1E+06	1,1E+06	1,1E+06	1,1E+06	1,1E+06
1.0E+02	1,1E+06	1,9E+06	1,6E+06	1,5E+06	1,4E+06	1,8E+06	1,6E+06	1,7E+06	1,7E+06	1,8E+06	1,7E+06	2,0E+06
6.3E+01	2,8E+06	2,7E+06	3,7E+06	2,1E+06	3,0E+06	1,6E+06	2,3E+06	4,3E+06	1,3E+06	2,8E+06	8,7E+05	4,1E+06
4.0E+01	3,5E+06	4,7E+06	4,5E+06	3,4E+06	7,0E+06	2,4E+06	4,4E+06	4,5E+06	1,6E+06	3,3E+06	1,5E+06	3,9E+06
2.5E+01	3,1E+06	6,8E+06	1,1E+07	1,4E+07	5,9E+06	1,5E+07	7,0E+06	9,2E+05	7,8E+06	9,5E+06	1,5E+06	8,8E+06
1.6E+01	8,0E+06	1,1E+07	7,3E+06	5,0E+06	9,5E+06	9,5E+06	9,3E+06	8,2E+06	6,7E+06	5,3E+06	8,8E+06	6,3E+06
1.0E+01	1,1E+07	1,5E+07	1,5E+07	1,4E+07	1,4E+07	1,5E+07	1,4E+07	1,5E+07	1,4E+07	1,4E+07	1,4E+07	1,3E+07
6.3E+00	1,7E+07	2,4E+07	2,2E+07	2,5E+07	2,3E+07	2,3E+07	1,9E+07	2,5E+07	2,3E+07	2,0E+07	2,1E+07	1,1E+07
4.0E+00	3,0E+07	4,1E+07	4,4E+07	3,7E+07	3,7E+07	3,2E+07	3,4E+07	3,8E+07	3,6E+07	2,7E+07	3,0E+07	3,4E+07
2.5E+00	5,8E+07	5,8E+07	5,7E+07	4,7E+07	6,5E+07	5,4E+07	5,2E+07	6,3E+07	5,3E+07	4,0E+07	5,6E+07	5,4E+07
1.6E+00	1,3E+08	1,0E+08	9,7E+07	8,2E+07	1,1E+08	1,0E+08	7,8E+07	8,3E+07	9,8E+07	9,5E+07	7,5E+07	1,1E+08
1.0E+00	4,5E+07	1,2E+08	1,1E+08	1,5E+08	2,7E+07	7,7E+07	1,5E+08	1,5E+08	2,0E+08	1,4E+08	1,3E+08	1,8E+08

Table S3. Standard deviation of the impedance measurements of eucalyptol obtained with the $(PAH/MWCNT)_5$-thin-film-based sensor at different frequencies.

Log_{10} Frequency (Hz)	Standard deviation of impedance (Ω)											
	0 ppm	68 ppm	109 ppm	205 ppm	315 ppm	410 ppm	506 ppm	602 ppm	712 ppm	807 ppm	903 ppm	999 ppm
1.0E+06	2,8E+01	2,6E+01	6,2E+00	7,4E+00	2,4E+01	5,6E+00	2,3E+01	4,8E+00	2,9E+01	9,0E-01	4,4E+00	2,6E+01
6.3E+05	4,3E+01	4,2E+01	5,9E+00	1,0E+01	4,2E+01	1,3E+01	4,0E+01	8,5E+00	4,1E+01	6,9E+00	8,2E+00	5,3E+01
4.0E+05	6,8E+01	7,0E+01	8,7E+00	1,6E+01	6,8E+01	2,9E+01	6,6E+01	1,5E+01	6,5E+01	1,3E+01	1,4E+01	9,1E+01
2.5E+05	1,1E+02	1,1E+02	1,3E+01	2,8E+01	1,1E+02	6,5E+01	1,1E+02	2,6E+01	1,0E+02	2,0E+01	2,3E+01	1,5E+02
1.6E+05	1,7E+02	1,8E+02	2,1E+01	4,7E+01	1,6E+02	1,4E+02	1,7E+02	4,8E+01	1,6E+02	3,3E+01	3,6E+01	2,4E+02
1.0E+05	2,7E+02	3,0E+02	3,4E+01	8,7E+01	2,1E+02	2,9E+02	2,7E+02	9,9E+01	2,5E+02	5,4E+01	6,1E+01	3,8E+02
6.3E+04	4,3E+02	4,7E+02	5,8E+01	1,7E+02	2,5E+02	5,2E+02	4,3E+02	2,2E+02	3,8E+02	8,8E+01	9,7E+01	6,1E+02
4.0E+04	6,8E+02	7,4E+02	8,6E+01	3,7E+02	2,3E+02	8,8E+02	6,7E+02	5,0E+02	5,6E+02	1,5E+02	1,5E+02	9,6E+02
2.5E+04	1,1E+03	7,6E+02	1,4E+02	8,1E+02	1,7E+02	1,4E+03	1,0E+03	1,0E+03	7,6E+02	2,2E+02	2,5E+02	1,5E+03
1.6E+04	1,7E+03	1,3E+03	2,2E+02	1,7E+03	9,9E+01	2,3E+03	1,6E+03	1,9E+03	8,6E+02	3,4E+02	3,9E+02	2,4E+03
1.0E+04	2,6E+03	2,6E+03	3,5E+02	3,1E+03	5,4E+00	3,6E+03	2,4E+03	3,4E+03	6,4E+02	5,9E+02	6,2E+02	3,8E+03
6.3E+03	3,8E+03	5,9E+03	5,7E+02	5,1E+03	5,1E+01	5,8E+03	3,4E+03	5,4E+03	4,8E+01	9,1E+02	9,6E+02	6,0E+03
4.0E+03	5,4E+03	9,7E+03	6,7E+02	7,7E+03	2,2E+02	9,1E+03	4,0E+03	8,7E+03	7,0E+02	1,4E+03	1,7E+03	9,4E+03
2.5E+03	5,8E+03	1,5E+04	1,3E+03	1,1E+04	3,2E+02	1,4E+04	4,0E+03	1,4E+04	2,0E+03	2,2E+03	2,3E+03	1,5E+04
1.6E+03	5,8E+03	2,4E+04	2,0E+03	1,3E+04	4,7E+02	2,2E+04	3,1E+03	2,2E+04	3,6E+03	3,6E+03	3,6E+03	2,3E+04
1.0E+03	3,2E+03	3,7E+04	3,0E+03	1,5E+04	1,2E+02	3,5E+04	3,6E+02	3,5E+04	5,4E+03	5,8E+03	6,0E+03	3,6E+04
6.3E+02	6,2E+03	6,2E+04	8,9E+02	3,1E+04	7,9E+03	4,2E+04	3,5E+02	5,2E+04	1,6E+04	1,7E+04	5,9E+03	5,1E+04
4.0E+02	9,9E+03	8,8E+04	2,7E+04	4,5E+04	3,9E+03	8,4E+04	6,4E+03	7,1E+04	5,5E+03	1,8E+04	1,4E+04	9,4E+04
2.5E+02	1,3E+05	1,1E+05	4,4E+04	1,4E+05	6,3E+04	9,8E+04	4,8E+04	1,6E+05	6,8E+04	9,4E+04	8,0E+04	2,8E+05
1.6E+02	0,0E+00	1,8E+05	5,0E+04	1,8E+05	1,7E+04	1,2E+05	1,7E+04	2,2E+05	5,0E+04	5,0E+04	4,1E+04	2,3E+05
1.0E+02	4,3E+05	3,3E+05	5,7E+05	1,1E+06	1,7E+04	6,0E+05	9,3E+05	8,2E+05	3,8E+05	2,0E+05	6,1E+05	4,8E+05
6.3E+01	5,8E+05	1,6E+06	1,2E+05	2,7E+05	1,2E+06	1,6E+06	1,6E+06	1,8E+06	9,0E+05	2,0E+05	2,8E+06	1,1E+06
4.0E+01	1,7E+04	5,2E+05	2,3E+06	1,1E+06	4,8E+05	2,3E+05	9,3E+05	2,0E+06	2,8E+05	8,3E+04	2,6E+06	2,8E+05
2.5E+01	1,1E+06	1,6E+06	1,7E+04	2,2E+06	2,0E+06	1,5E+05	1,8E+06	3,0E+06	5,0E+05	1,4E+06	4,6E+05	5,0E+06
1.6E+01	6,7E+05	1,3E+06	2,0E+06	3,5E+06	3,3E+05	1,7E+05	1,3E+06	3,3E+05	2,5E+06	6,3E+06	5,7E+04	1,8E+06
1.0E+01	6,7E+05	1,2E+06	2,7E+06	3,0E+06	2,3E+06	6,7E+05	5,0E+05	3,2E+06	2,7E+06	3,3E+05	3,0E+06	4,0E+06
6.3E+00	1,7E+05	4,7E+06	6,0E+06	4,3E+06	4,2E+06	8,8E+06	1,7E+05	6,0E+06	1,7E+06	1,5E+06	6,6E+06	5,0E+06
4.0E+00	3,2E+06	2,8E+06	6,3E+06	7,7E+06	6,0E+06	1,3E+06	0,0E+00	8,3E+06	1,0E+07	1,8E+06	9,0E+06	1,0E+07
2.5E+00	4,2E+06	1,3E+07	2,0E+07	1,2E+07	3,7E+06	1,0E+06	8,2E+06	1,5E+07	1,9E+07	2,3E+06	7,2E+06	5,2E+06
1.6E+00	1,2E+07	1,5E+07	1,5E+07	1,3E+07	1,0E+07	1,3E+07	1,0E+07	2,3E+07	1,3E+07	3,3E+06	1,4E+07	2,0E+07
1.0E+00	8,3E+06	3,0E+07	2,3E+07	8,2E+07	3,2E+07	1,2E+07	1,3E+07	2,8E+07	2,5E+07	3,3E+06	4,0E+07	3,2E+07

Table S4. Standard deviation of the impedance measurements of eucalyptol obtained with the (PAH/MWCNT-COOH)$_5$-thin-film-based sensor at different frequencies.

Log_{10} Frequency (Hz)	Standard deviation of impedance (Ω)											
	0 ppm	68 ppm	109 ppm	205 ppm	315 ppm	410 ppm	506 ppm	602 ppm	712 ppm	807 ppm	903 ppm	999 ppm
1.0E+06	1,6E+00	3,6E+00	4,3E+00	2,7E+01	2,7E+01	2,9E+01	2,8E+01	5,6E+01	1,1E+00	2,7E+01	2,8E+01	2,3E+01
6.3E+05	7,1E-01	1,4E-01	6,2E+00	4,7E+01	4,8E+01	5,0E+01	4,7E+01	9,5E+01	8,7E-01	4,5E+01	4,5E+01	3,0E+01
4.0E+05	2,1E-01	3,1E+00	9,5E+00	7,8E+01	7,9E+01	8,2E+01	7,7E+01	1,5E+02	5,1E-01	7,5E+01	7,4E+01	3,4E+01
2.5E+05	1,3E+00	6,0E+00	1,5E+01	1,3E+02	1,3E+02	1,3E+02	1,2E+02	2,5E+02	6,5E-01	1,2E+02	1,2E+02	3,1E+01
1.6E+05	1,7E+00	1,0E+01	2,6E+01	2,0E+02	2,0E+02	2,1E+02	2,0E+02	3,9E+02	1,8E+00	1,9E+02	1,9E+02	2,2E+01
1.0E+05	3,9E+00	2,4E+01	4,4E+01	3,3E+02	3,3E+02	3,5E+02	3,2E+02	6,3E+02	7,1E+00	3,2E+02	3,1E+02	1,5E+01
6.3E+04	5,3E+00	4,1E+01	7,3E+01	5,3E+02	5,2E+02	5,5E+02	5,1E+02	9,9E+02	1,6E+01	5,0E+02	4,9E+02	6,8E+00
4.0E+04	9,5E+00	6,5E+01	1,1E+02	8,3E+02	8,3E+02	8,8E+02	8,1E+02	1,6E+03	2,9E+01	8,0E+02	7,8E+02	2,7E+00
2.5E+04	2,1E+00	1,0E+02	1,9E+02	1,3E+03	1,3E+03	1,4E+03	1,3E+03	2,5E+03	4,6E+01	1,3E+03	1,2E+03	5,8E+00
1.6E+04	2,3E+01	1,3E+02	2,9E+02	2,1E+03	2,1E+03	2,2E+03	2,0E+03	3,9E+03	7,9E+01	2,0E+03	2,0E+03	1,4E+01
1.0E+04	1,3E+02	1,6E+02	5,0E+02	3,4E+03	3,3E+03	3,5E+03	3,3E+03	6,1E+03	1,5E+02	3,2E+03	3,1E+03	3,8E+00
6.3E+03	6,7E+02	1,4E+01	7,9E+02	5,2E+03	5,2E+03	5,5E+03	5,1E+03	9,5E+03	2,2E+02	5,1E+03	5,0E+03	1,6E+01
4.0E+03	2,4E+03	9,0E+02	1,2E+03	8,3E+03	8,3E+03	8,9E+03	8,2E+03	1,5E+04	2,2E+02	8,2E+03	8,0E+03	1,9E+02
2.5E+03	6,2E+03	3,9E+03	1,9E+03	1,3E+04	1,3E+04	1,4E+04	1,3E+04	2,2E+04	6,5E+02	1,2E+04	1,2E+04	3,1E+01
1.6E+03	1,3E+04	1,0E+04	3,0E+03	2,1E+04	2,0E+04	2,1E+04	1,9E+04	3,3E+04	9,1E+02	2,0E+04	1,9E+04	2,8E+01
1.0E+03	2,3E+04	2,1E+04	5,7E+03	3,3E+04	3,4E+04	3,4E+04	3,0E+04	4,7E+04	1,2E+03	3,1E+04	3,0E+04	6,4E+02
6.3E+02	4,2E+04	3,8E+04	1,0E+04	4,9E+04	4,0E+04	5,6E+04	3,9E+04	6,4E+04	5,4E+03	4,3E+04	4,1E+04	6,1E+03
4.0E+02	4,5E+04	4,6E+04	8,2E+03	7,1E+04	1,1E+05	1,0E+05	4,8E+04	1,0E+05	1,9E+04	8,5E+04	8,6E+04	1,1E+04
2.5E+02	5,0E+04	2,3E+04	5,2E+04	6,1E+04	1,0E+05	1,0E+05	1,3E+04	1,3E+05	9,2E+04	9,2E+03	9,3E+04	8,4E+04
1.6E+02	1,0E+05	1,5E+05	0,0E+00	2,2E+05	1,8E+05	2,0E+05	3,3E+04	1,7E+05	0,0E+00	1,8E+05	2,0E+05	1,7E+04
1.0E+02	6,2E+05	1,2E+05	5,5E+05	5,5E+05	5,0E+04	1,0E+05	2,0E+05	0,0E+00	1,2E+05	6,7E+04	5,2E+05	1,8E+05
6.3E+01	1,5E+06	5,8E+05	1,6E+06	5,7E+05	2,7E+05	1,1E+06	1,1E+06	9,3E+05	8,7E+05	7,3E+05	2,7E+05	1,3E+05
4.0E+01	1,3E+06	1,1E+06	1,4E+06	1,3E+06	2,2E+06	1,3E+05	6,5E+05	2,2E+06	2,5E+05	9,7E+05	1,6E+06	9,5E+05
2.5E+01	1,7E+04	4,7E+05	1,7E+05	3,5E+05	2,4E+06	4,8E+05	5,7E+05	3,5E+06	3,5E+05	2,2E+05	1,4E+06	1,2E+06
1.6E+01	1,7E+06	5,0E+05	3,3E+06	4,2E+06	1,2E+06	1,3E+06	1,5E+06	4,8E+06	3,3E+05	3,5E+06	1,7E+05	5,7E+06
1.0E+01	3,3E+05	2,3E+06	2,3E+06	3,3E+05	1,7E+05	5,0E+05	0,0E+00	2,5E+06	1,7E+05	1,7E+05	3,3E+05	3,3E+05
6.3E+00	1,8E+06	1,0E+06	5,0E+06	3,2E+06	5,0E+05	8,3E+05	3,3E+06	4,7E+06	1,5E+06	4,3E+06	1,7E+05	8,3E+05
4.0E+00	5,0E+05	4,3E+06	8,3E+06	1,8E+06	3,3E+05	5,0E+05	4,3E+06	2,3E+07	1,5E+07	1,7E+05	1,3E+06	7,8E+06
2.5E+00	5,8E+06	8,8E+06	2,2E+07	5,2E+06	4,2E+06	8,5E+06	3,3E+05	1,5E+07	2,3E+06	1,6E+07	4,3E+06	1,4E+07
1.6E+00	1,0E+07	1,3E+07	1,7E+06	1,7E+06	5,0E+06	5,0E+06	5,0E+06	1,5E+07	1,0E+07	3,8E+07	6,7E+06	2,0E+07
1.0E+00	3,3E+06	1,5E+07	2,7E+07	3,3E+06	6,7E+06	1,3E+08	2,7E+07	1,7E+07	3,3E+06	5,0E+06	9,0E+07	3,7E+07

Table S5. Standard deviation of the impedance measurements of eucalyptol obtained with the ZnO (50% O_2)-thin-film-based sensor at different frequencies.

Log_{10} Frequency (Hz)	Standard deviation of impedance (Ω)											
	0 ppm	68 ppm	109 ppm	205 ppm	315 ppm	410 ppm	506 ppm	602 ppm	712 ppm	807 ppm	903 ppm	999 ppm
1.0E+06	2,1E+00	2,1E+00	2,1E+00	2,1E+00	2,1E+00	2,0E+00	2,1E+00	2,0E+00	2,0E+00	2,0E+00	2,0E+00	2,0E+00
6.3E+05	2,1E+00	2,1E+00	2,1E+00	2,1E+00	2,0E+00	2,0E+00	2,0E+00	2,0E+00	2,0E+00	2,0E+00	2,1E+00	2,0E+00
4.0E+05	2,1E+00	2,1E+00	2,1E+00	2,1E+00	2,1E+00	2,0E+00	2,0E+00	2,0E+00	2,0E+00	2,0E+00	2,0E+00	2,0E+00
2.5E+05	2,2E+00	2,1E+00	2,1E+00	2,1E+00	2,1E+00	2,1E+00	2,0E+00	2,0E+00	2,0E+00	2,0E+00	2,0E+00	2,0E+00
1.6E+05	2,2E+00	2,1E+00	2,1E+00	2,1E+00	2,1E+00	2,1E+00	2,0E+00	2,0E+00	2,0E+00	2,0E+00	2,0E+00	2,0E+00
1.0E+05	2,2E+00	2,1E+00	2,1E+00	2,1E+00	2,1E+00	2,1E+00	2,1E+00	2,0E+00	2,0E+00	2,0E+00	2,0E+00	2,0E+00
6.3E+04	2,2E+00	2,2E+00	2,1E+00	2,1E+00	2,1E+00	2,1E+00	2,1E+00	2,0E+00	2,0E+00	2,0E+00	2,0E+00	2,0E+00
4.0E+04	2,2E+00	2,2E+00	2,1E+00	2,1E+00	2,1E+00	2,1E+00	2,1E+00	2,0E+00	2,0E+00	2,0E+00	2,0E+00	2,0E+00
2.5E+04	2,2E+00	2,2E+00	2,1E+00	2,1E+00	2,1E+00	2,1E+00	2,1E+00	2,1E+00	2,0E+00	2,0E+00	2,0E+00	2,0E+00
1.6E+04	2,2E+00	2,2E+00	2,1E+00	2,1E+00	2,1E+00	2,1E+00	2,1E+00	2,1E+00	2,0E+00	2,0E+00	2,0E+00	2,0E+00
1.0E+04	2,2E+00	2,2E+00	2,1E+00	2,1E+00	2,1E+00	2,1E+00	2,1E+00	2,1E+00	2,0E+00	2,0E+00	2,0E+00	2,0E+00
6.3E+03	2,2E+00	2,2E+00	2,1E+00	2,1E+00	2,1E+00	2,1E+00	2,1E+00	2,1E+00	2,0E+00	2,0E+00	2,0E+00	2,0E+00
4.0E+03	2,2E+00	2,2E+00	2,1E+00	2,1E+00	2,1E+00	2,1E+00	2,1E+00	2,1E+00	2,0E+00	2,0E+00	2,0E+00	2,0E+00
2.5E+03	2,2E+00	2,2E+00	2,1E+00	2,1E+00	2,1E+00	2,1E+00	2,1E+00	2,1E+00	2,0E+00	2,0E+00	2,0E+00	2,0E+00
1.6E+03	2,2E+00	2,2E+00	2,1E+00	2,1E+00	2,1E+00	2,1E+00	2,1E+00	2,1E+00	2,0E+00	2,0E+00	2,0E+00	2,0E+00

1.0E+03	2,2E+00	2,2E+00	2,1E+00	2,1E+00	2,1E+00	2,1E+00	2,1E+00	2,1E+00	2,0E+00	2,0E+00	2,0E+00	2,0E+00
6.3E+02	2,2E+00	2,2E+00	2,1E+00	2,1E+00	2,1E+00	2,1E+00	2,1E+00	2,1E+00	2,0E+00	2,0E+00	2,0E+00	2,0E+00
4.0E+02	2,2E+00	2,2E+00	2,1E+00	2,1E+00	2,1E+00	2,1E+00	2,1E+00	2,1E+00	2,0E+00	2,0E+00	2,0E+00	2,0E+00
2.5E+02	2,2E+00	2,2E+00	2,1E+00	2,1E+00	2,1E+00	2,1E+00	2,1E+00	2,1E+00	2,0E+00	2,0E+00	2,0E+00	2,0E+00
1.6E+02	2,2E+00	2,2E+00	2,1E+00	2,1E+00	2,1E+00	2,1E+00	2,1E+00	2,1E+00	2,0E+00	2,0E+00	2,0E+00	2,0E+00
1.0E+02	2,2E+00	2,2E+00	2,1E+00	2,1E+00	2,1E+00	2,1E+00	2,1E+00	2,1E+00	2,0E+00	2,0E+00	2,0E+00	2,0E+00
6.3E+01	2,2E+00	2,2E+00	2,1E+00	2,1E+00	2,1E+00	2,1E+00	2,1E+00	2,1E+00	2,0E+00	2,0E+00	2,0E+00	2,0E+00
4.0E+01	2,2E+00	2,2E+00	2,1E+00	2,1E+00	2,1E+00	2,1E+00	2,1E+00	2,1E+00	2,0E+00	2,0E+00	2,0E+00	2,0E+00
2.5E+01	2,2E+00	2,2E+00	2,1E+00	2,1E+00	2,1E+00	2,1E+00	2,1E+00	2,1E+00	2,0E+00	2,0E+00	2,0E+00	2,0E+00
1.6E+01	2,2E+00	2,2E+00	2,1E+00	2,1E+00	2,1E+00	2,1E+00	2,1E+00	2,1E+00	2,0E+00	2,0E+00	2,0E+00	2,0E+00
1.0E+01	2,2E+00	2,2E+00	2,1E+00	2,1E+00	2,1E+00	2,1E+00	2,1E+00	2,1E+00	2,0E+00	2,0E+00	2,0E+00	2,0E+00
6.3E+00	2,2E+00	2,2E+00	2,1E+00	2,1E+00	2,1E+00	2,1E+00	2,1E+00	2,1E+00	2,0E+00	2,0E+00	2,0E+00	2,0E+00
4.0E+00	2,2E+00	2,2E+00	2,1E+00	2,1E+00	2,1E+00	2,1E+00	2,1E+00	2,1E+00	2,0E+00	2,0E+00	2,0E+00	2,0E+00
2.5E+00	2,2E+00	2,2E+00	2,1E+00	2,1E+00	2,1E+00	2,1E+00	2,1E+00	2,1E+00	2,0E+00	2,0E+00	2,0E+00	2,0E+00
1.6E+00	2,2E+00	2,2E+00	2,1E+00	2,1E+00	2,1E+00	2,1E+00	2,1E+00	2,1E+00	2,0E+00	2,0E+00	2,0E+00	2,0E+00
1.0E+00	2,2E+00	2,2E+00	2,1E+00	2,1E+00	2,1E+00	2,1E+00	2,1E+00	2,1E+00	2,0E+00	2,0E+00	2,0E+00	2,0E+00

Table S6. Standard deviation of the impedance measurements of eucalyptol obtained with the ZnO (100% O_2)-thin-film-based sensor at different frequencies.

Log_{10} Frequency (Hz)	Standard deviation of impedance (Ω)											
	0 ppm	68 ppm	109 ppm	205 ppm	315 ppm	410 ppm	506 ppm	602 ppm	712 ppm	807 ppm	903 ppm	999 ppm
1.0E+06	4,7E-01	1,2E+02	1,2E+02	6,0E+00	3,5E+00	2,8E+00	4,4E+00	3,0E+00	3,9E+00	2,1E+00	2,5E+00	2,8E+00
6.3E+05	7,3E+00	1,9E+02	2,1E+02	8,5E+00	1,2E+01	1,1E+01	1,3E+01	1,1E+01	5,8E+00	8,7E+00	8,5E+00	9,2E+00
4.0E+05	2,7E+01	3,0E+02	3,5E+02	3,1E+01	3,0E+01	2,8E+01	3,2E+01	2,8E+01	2,2E+01	2,6E+01	2,6E+01	2,7E+01
2.5E+05	7,0E+01	4,5E+02	5,8E+02	7,2E+01	6,8E+01	6,4E+01	7,0E+01	6,3E+01	5,3E+01	5,9E+01	5,9E+01	6,1E+01
1.6E+05	1,7E+02	6,7E+02	9,5E+02	1,5E+02	1,5E+02	1,4E+02	1,5E+02	1,4E+02	1,2E+02	1,3E+02	1,3E+02	1,3E+02
1.0E+05	3,8E+02	9,7E+02	1,6E+03	3,2E+02	3,0E+02	2,9E+02	3,1E+02	2,8E+02	2,5E+02	2,7E+02	2,6E+02	2,7E+02
6.3E+04	8,8E+02	1,5E+03	2,7E+03	6,8E+02	6,5E+02	6,2E+02	6,4E+02	5,9E+02	5,4E+02	5,6E+02	5,5E+02	5,5E+02
4.0E+04	2,2E+03	1,8E+03	4,6E+03	1,5E+03	1,5E+03	1,4E+03	1,4E+03	1,3E+03	1,2E+03	1,2E+03	1,2E+03	1,2E+03
2.5E+04	5,8E+03	8,3E+02	8,7E+03	3,8E+03	3,7E+03	3,5E+03	3,5E+03	3,2E+03	3,0E+03	2,9E+03	2,8E+03	2,8E+03
1.6E+04	1,6E+04	4,7E+03	2,2E+04	1,0E+04	9,9E+03	9,6E+03	9,2E+03	8,6E+03	8,1E+03	7,8E+03	7,4E+03	7,1E+03
1.0E+04	4,2E+04	2,2E+04	4,6E+04	2,8E+04	2,7E+04	2,6E+04	2,5E+04	2,4E+04	2,2E+04	2,1E+04	2,0E+04	1,9E+04
6.3E+03	1,0E+05	6,7E+04	9,8E+04	7,0E+04	6,8E+04	6,7E+04	6,4E+04	6,1E+04	5,9E+04	5,6E+04	5,4E+04	5,2E+04
4.0E+03	2,1E+05	1,6E+05	2,0E+05	1,5E+05	1,5E+05	1,5E+05	1,4E+05	1,4E+05	1,4E+05	1,3E+05	1,3E+05	1,2E+05
2.5E+03	3,8E+05	3,0E+05	3,6E+05	3,0E+05	2,9E+05	2,9E+05	2,8E+05	2,7E+05	2,7E+05	2,6E+05	2,6E+05	2,5E+05
1.6E+03	6,2E+05	5,2E+05	6,0E+05	5,1E+05	5,0E+05	4,9E+05	4,8E+05	4,7E+05	4,6E+05	4,6E+05	4,5E+05	4,4E+05
1.0E+03	9,4E+05	8,1E+05	8,9E+05	7,8E+05	7,6E+05	7,5E+05	7,3E+05	7,2E+05	7,1E+05	7,0E+05	6,8E+05	6,7E+05
6.3E+02	1,3E+06	1,1E+06	1,2E+06	1,1E+06	1,0E+06	1,0E+06	9,8E+05	9,6E+05	9,4E+05	9,3E+05	9,1E+05	8,9E+05
4.0E+02	1,5E+06	1,4E+06	1,4E+06	1,3E+06	1,3E+06	1,2E+06	1,2E+06	1,1E+06	1,1E+06	1,1E+06	1,1E+06	1,0E+06
2.5E+02	1,7E+06	1,6E+06	1,5E+06	1,4E+06	1,4E+06	1,3E+06	1,3E+06	1,2E+06	1,2E+06	1,2E+06	1,2E+06	1,1E+06
1.6E+02	1,8E+06	1,7E+06	1,6E+06	1,5E+06	1,4E+06	1,4E+06	1,3E+06	1,3E+06	1,3E+06	1,2E+06	1,2E+06	1,2E+06
1.0E+02	1,9E+06	1,7E+06	1,6E+06	1,6E+06	1,5E+06	1,4E+06	1,4E+06	1,3E+06	1,3E+06	1,3E+06	1,2E+06	1,2E+06
6.3E+01	1,9E+06	1,7E+06	1,7E+06	1,6E+06	1,5E+06	1,4E+06	1,4E+06	1,3E+06	1,3E+06	1,3E+06	1,2E+06	1,2E+06
4.0E+01	1,9E+06	1,8E+06	1,7E+06	1,6E+06	1,5E+06	1,4E+06	1,4E+06	1,3E+06	1,3E+06	1,3E+06	1,2E+06	1,2E+06
2.5E+01	1,9E+06	1,7E+06	1,7E+06	1,6E+06	1,5E+06	1,4E+06	1,4E+06	1,3E+06	1,3E+06	1,3E+06	1,3E+06	1,2E+06
1.6E+01	1,9E+06	1,8E+06	1,7E+06	1,6E+06	1,5E+06	1,4E+06	1,4E+06	1,3E+06	1,3E+06	1,3E+06	1,3E+06	1,2E+06
1.0E+01	1,9E+06	1,8E+06	1,7E+06	1,6E+06	1,5E+06	1,4E+06	1,4E+06	1,3E+06	1,3E+06	1,3E+06	1,3E+06	1,2E+06
6.3E+00	1,9E+06	1,8E+06	1,7E+06	1,6E+06	1,5E+06	1,4E+06	1,4E+06	1,3E+06	1,3E+06	1,3E+06	1,3E+06	1,2E+06
4.0E+00	1,9E+06	1,8E+06	1,7E+06	1,6E+06	1,5E+06	1,4E+06	1,4E+06	1,3E+06	1,3E+06	1,3E+06	1,3E+06	1,2E+06
2.5E+00	1,9E+06	1,8E+06	1,7E+06	1,6E+06	1,5E+06	1,4E+06	1,4E+06	1,3E+06	1,3E+06	1,3E+06	1,3E+06	1,2E+06
1.6E+00	1,9E+06	1,8E+06	1,7E+06	1,6E+06	1,5E+06	1,4E+06	1,4E+06	1,3E+06	1,3E+06	1,3E+06	1,2E+06	1,2E+06
1.0E+00	1,9E+06	1,8E+06	1,7E+06	1,6E+06	1,5E+06	1,4E+06	1,4E+06	1,3E+06	1,3E+06	1,3E+06	1,3E+06	1,2E+06

Table S7. Standard deviation of the impedance measurements of eucalyptol obtained with the TiO_2 (50% O_2)-thin-film-based sensor at different frequencies.

Log10 Frequency (Hz)	Standard deviation of impedance (Ω)											
	0 ppm	68 ppm	109 ppm	205 ppm	315 ppm	410 ppm	506 ppm	602 ppm	712 ppm	807 ppm	903 ppm	999 ppm
1.0E+06	2.1E+01	2.1E+01	2.3E+01	1.0E+01	2.4E+01	2.4E+01	3.3E+01	1.3E+01	5.1E+00	6.2E+00	3.0E+01	3.0E+01
6.3E+05	2.4E+00	2.6E+00	7.5E+00	1.8E+01	4.0E+01	4.1E+01	5.6E+01	3.1E+01	8.6E+00	1.1E+01	4.9E+01	5.0E+01
4.0E+05	1.7E+01	1.6E+01	7.0E+00	3.2E+01	5.5E+01	6.7E+01	9.1E+01	6.7E+01	1.4E+01	1.8E+01	8.0E+01	8.1E+01
2.5E+05	4.3E+01	4.2E+01	2.6E+01	5.5E+01	8.4E+01	1.1E+02	1.5E+02	1.3E+02	2.3E+01	3.0E+01	1.3E+02	1.3E+02
1.6E+05	8.3E+01	8.2E+01	5.6E+01	1.0E+02	1.2E+02	1.7E+02	2.3E+02	2.2E+02	3.7E+01	4.8E+01	2.1E+02	2.1E+02
1.0E+05	1.6E+02	1.5E+02	1.1E+02	1.9E+02	2.0E+02	2.8E+02	3.8E+02	3.7E+02	6.1E+01	7.8E+01	3.4E+02	3.5E+02
6.3E+04	2.7E+02	2.7E+02	1.9E+02	3.1E+02	3.6E+02	4.4E+02	6.0E+02	5.9E+02	9.6E+01	1.2E+02	5.4E+02	5.5E+02
4.0E+04	4.6E+02	4.5E+02	3.4E+02	2.9E+02	6.3E+02	7.0E+02	9.4E+02	9.3E+02	1.5E+02	2.0E+02	8.7E+02	8.8E+02
2.5E+04	7.6E+02	7.4E+02	5.8E+02	4.7E+02	1.1E+03	1.1E+03	1.5E+03	1.5E+03	2.4E+02	3.1E+02	1.4E+03	1.4E+03
1.6E+04	1.2E+03	1.1E+03	9.7E+02	8.4E+02	1.8E+03	1.7E+03	2.3E+03	2.3E+03	3.7E+02	4.8E+02	2.1E+03	2.2E+03
1.0E+04	1.9E+03	1.8E+03	1.8E+03	1.6E+03	2.8E+03	2.7E+03	3.7E+03	3.7E+03	5.9E+02	7.6E+02	3.4E+03	3.5E+03
6.3E+03	2.9E+03	2.8E+03	3.6E+03	3.0E+03	4.7E+03	4.3E+03	5.8E+03	5.8E+03	9.0E+02	1.2E+03	5.4E+03	5.5E+03
4.0E+03	4.6E+03	4.3E+03	7.2E+03	5.7E+03	7.6E+03	7.0E+03	9.1E+03	9.3E+03	1.6E+03	1.7E+03	8.2E+03	8.6E+03
2.5E+03	7.1E+03	6.7E+03	1.4E+04	1.0E+04	1.4E+04	1.1E+04	1.4E+04	1.4E+04	2.2E+03	2.9E+03	1.3E+04	1.4E+04
1.6E+03	1.1E+04	1.0E+04	2.4E+04	1.7E+04	2.3E+04	1.6E+04	2.2E+04	2.2E+04	3.3E+03	4.4E+03	2.1E+04	2.1E+04
1.0E+03	1.6E+04	1.6E+04	3.9E+04	2.9E+04	3.7E+04	2.7E+04	3.5E+04	3.5E+04	5.4E+03	8.4E+03	3.3E+04	3.2E+04
6.3E+02	2.7E+04	2.5E+04	6.5E+04	4.2E+04	5.5E+04	4.5E+04	5.3E+04	5.2E+04	3.3E+03	1.2E+04	5.2E+04	5.3E+04
4.0E+02	3.6E+04	4.3E+04	9.3E+04	6.7E+04	7.3E+04	7.9E+04	8.0E+04	7.0E+04	2.1E+04	3.6E+03	8.3E+04	6.0E+04
2.5E+02	1.0E+05	4.1E+04	1.6E+05	2.5E+05	1.5E+05	1.2E+05	1.3E+05	6.0E+04	4.8E+04	6.6E+04	1.1E+05	7.4E+04
1.6E+02	1.2E+05	1.1E+05	2.7E+05	2.1E+05	2.4E+05	1.9E+05	2.1E+05	2.1E+05	4.7E+04	7.2E+04	1.9E+05	1.9E+05
1.0E+02	8.4E+04	1.2E+05	5.2E+05	8.3E+04	3.8E+05	3.8E+05	3.0E+05	2.8E+05	1.4E+05	1.5E+05	1.1E+05	4.0E+05
6.3E+01	6.1E+05	6.8E+05	1.4E+06	2.3E+05	1.1E+06	1.1E+05	2.6E+05	7.3E+05	1.1E+06	1.1E+06	1.8E+06	5.9E+05
4.0E+01	1.3E+06	8.6E+05	1.2E+06	1.9E+06	8.0E+05	5.6E+05	2.0E+06	1.7E+06	1.0E+06	1.4E+06	3.1E+04	2.5E+06
2.5E+01	3.5E+04	9.6E+05	2.6E+06	3.1E+06	2.7E+05	8.1E+05	1.5E+06	1.7E+06	4.1E+05	9.8E+05	4.6E+05	7.0E+05
1.6E+01	9.7E+05	3.5E+05	5.7E+05	3.7E+06	3.2E+06	7.6E+04	1.6E+06	3.4E+06	9.8E+05	5.9E+06	1.2E+06	2.8E+06
1.0E+01	2.3E+05	3.7E+05	3.8E+06	3.9E+06	3.3E+06	2.0E+06	2.6E+06	2.9E+06	2.6E+06	3.1E+06	4.2E+05	6.8E+05
6.3E+00	2.1E+06	4.9E+04	5.1E+06	5.9E+06	3.5E+06	3.6E+06	2.6E+06	6.0E+06	6.0E+06	6.3E+06	1.6E+05	8.8E+05
4.0E+00	3.3E+06	1.2E+06	1.1E+07	8.9E+06	5.3E+06	6.9E+06	3.2E+06	4.0E+06	9.1E+06	7.9E+06	2.6E+06	3.3E+06
2.5E+00	6.4E+05	3.0E+06	1.1E+07	1.5E+07	2.7E+07	1.4E+07	7.8E+06	9.9E+06	1.7E+07	9.7E+05	4.5E+06	7.2E+06
1.6E+00	1.0E+07	3.3E+06	1.9E+07	2.1E+07	2.0E+07	5.3E+06	1.5E+07	1.7E+07	1.2E+07	1.2E+07	2.7E+07	1.6E+07
1.0E+00	1.4E+07	1.1E+06	3.4E+07	3.3E+07	2.9E+08	3.5E+08	7.7E+07	1.9E+07	6.4E+07	1.6E+07	1.0E+07	1.6E+08

Table S8. Standard deviation of the impedance measurements of eucalyptol obtained with the TiO_2 (100% O_2)-thin-film-based sensor at different frequencies.

Log10 Frequency (Hz)	Standard deviation of impedance (Ω)											
	0 ppm	68 ppm	109 ppm	205 ppm	315 ppm	410 ppm	506 ppm	602 ppm	712 ppm	807 ppm	903 ppm	999 ppm
1.0E+06	1.0E+02	6.7E+01	3.3E+02	3.1E+02	9.7E+01	3.9E+02	2.4E+02	3.7E+02	1.5E+02	2.8E+02	3.1E+02	1.6E+02
6.3E+05	1.6E+02	3.4E+02	1.1E+02	1.1E+02	2.4E+02	4.0E+02	1.4E+02	1.5E+02	1.3E+02	1.4E+02	3.2E+02	2.6E+02
4.0E+05	2.6E+02	2.6E+02	1.4E+02	1.8E+02	3.0E+02	1.3E+02	3.7E+02	5.3E+01	2.0E+02	3.9E+02	1.3E+02	1.6E+02
2.5E+05	1.8E+02	1.2E+02	1.7E+02	1.3E+02	3.1E+02	2.5E+02	6.6E+01	1.3E+02	2.8E+02	3.9E+02	1.3E+02	1.9E+02
1.6E+05	3.0E+02	3.6E+02	4.2E+02	4.7E+01	2.2E+02	3.7E+02	2.5E+02	1.2E+02	2.7E+02	1.7E+02	2.4E+02	1.5E+02
1.0E+05	3.6E+02	2.1E+02	5.7E+01	3.0E+02	2.4E+02	3.2E+02	2.5E+02	3.9E+01	4.2E+02	3.7E+02	2.2E+02	7.8E+01
6.3E+04	2.0E+02	4.2E+02	4.0E+02	3.3E+02	2.9E+02	4.0E+02	9.2E+01	3.5E+02	4.1E+02	2.9E+02	1.6E+02	5.2E+01
4.0E+04	2.9E+02	4.2E+02	1.2E+02	3.3E+02	2.7E+02	1.2E+02	3.1E+02	4.0E+02	3.6E+02	9.5E+01	1.3E+02	2.2E+02
2.5E+04	2.4E+02	3.5E+02	6.0E+01	4.1E+02	8.6E+01	1.0E+02	2.8E+02	6.3E+01	3.6E+02	4.1E+02	4.2E+02	1.9E+02
1.6E+04	6.1E+01	1.2E+02	7.2E+01	3.6E+02	1.5E+02	2.0E+02	2.6E+02	2.9E+02	4.6E+01	8.3E+01	3.2E+02	2.9E+02
1.0E+04	2.7E+02	3.2E+02	3.5E+01	2.9E+02	3.7E+02	6.5E+01	2.5E+02	2.5E+02	3.4E+02	1.2E+02	1.3E+02	2.2E+02
6.3E+03	1.6E+02	4.2E+02	3.2E+01	9.9E+01	1.6E+02	3.3E+02	1.5E+02	2.5E+02	3.2E+02	1.1E+02	2.1E+02	3.9E+02
4.0E+03	2.6E+02	3.9E+02	3.9E+02	2.0E+02	4.7E+01	4.1E+02	2.9E+02	1.5E+02	1.2E+02	1.6E+02	2.5E+02	1.2E+02
2.5E+03	2.3E+02	6.0E+01	2.8E+02	1.0E+02	2.3E+02	4.0E+02	2.8E+02	1.9E+02	1.7E+02	1.3E+02	1.2E+02	1.9E+02
1.6E+03	3.6E+02	1.3E+02	3.0E+02	3.5E+02	9.1E+01	1.9E+02	4.0E+02	2.2E+02	1.0E+02	1.3E+02	1.3E+02	9.2E+01

1.0E+03	1.8E+02	4.8E+01	3.5E+02	3.5E+02	9.6E+01	3.3E+02	7.8E+01	1.5E+02	3.7E+02	3.8E+02	1.1E+02	1.9E+02
6.3E+02	1.7E+02	2.0E+02	4.9E+01	2.2E+02	1.1E+02	3.2E+02	4.1E+02	1.9E+02	1.2E+02	3.7E+02	2.0E+02	1.3E+02
4.0E+02	3.8E+02	1.7E+02	4.4E+01	3.7E+02	3.1E+02	3.0E+02	2.4E+02	2.3E+02	5.8E+01	3.4E+02	3.2E+02	3.8E+02
2.5E+02	1.8E+02	1.4E+02	3.1E+02	1.4E+02	2.9E+02	3.8E+02	2.6E+02	3.7E+02	1.2E+02	3.7E+02	8.4E+01	1.2E+02
1.6E+02	1.2E+02	2.8E+02	1.3E+02	2.4E+02	1.5E+02	2.8E+02	6.3E+01	2.2E+02	2.5E+02	1.0E+02	1.1E+02	3.4E+02
1.0E+02	3.7E+02	3.5E+02	3.7E+02	3.8E+01	3.4E+02	2.2E+02	2.0E+02	4.1E+02	1.8E+02	8.0E+01	4.1E+01	4.2E+02
6.3E+01	9.7E+01	9.9E+01	2.5E+02	3.9E+02	1.3E+02	6.1E+01	3.3E+01	7.0E+01	1.5E+02	3.1E+01	4.0E+02	4.2E+01
4.0E+01	3.7E+02	1.8E+02	9.5E+01	9.7E+01	3.9E+02	4.2E+02	3.8E+02	3.4E+02	2.8E+02	7.0E+01	1.7E+02	4.0E+02
2.5E+01	3.2E+02	4.0E+02	2.5E+02	3.9E+02	3.1E+02	1.4E+02	3.6E+02	2.2E+02	4.1E+02	2.7E+02	3.9E+02	4.2E+02
1.6E+01	5.8E+01	2.9E+02	8.9E+01	2.4E+02	3.1E+02	2.7E+02	2.8E+02	5.9E+01	4.0E+02	2.5E+02	1.0E+02	1.4E+02
1.0E+01	2.4E+02	3.8E+02	1.5E+02	4.1E+02	3.1E+02	3.5E+02	1.4E+02	2.1E+02	1.7E+02	1.2E+02	1.5E+02	3.0E+02
6.3E+00	3.8E+02	2.1E+02	6.2E+01	2.6E+02	9.7E+01	1.5E+02	2.2E+02	4.2E+02	3.2E+02	2.7E+02	3.3E+02	1.1E+02
4.0E+00	2.1E+02	2.9E+02	8.9E+01	3.4E+02	1.4E+02	3.2E+02	2.2E+02	3.5E+02	2.1E+02	7.1E+01	1.8E+02	1.2E+02
2.5E+00	4.1E+02	2.8E+02	3.4E+02	3.3E+02	2.2E+02	2.6E+02	4.0E+02	6.5E+01	1.2E+02	3.7E+02	3.8E+02	8.3E+01
1.6E+00	1.1E+02	9.8E+01	1.3E+02	4.0E+02	1.8E+02	3.9E+02	3.9E+02	3.9E+02	2.5E+02	1.5E+02	1.1E+02	4.0E+02
1.0E+00	9.5E+01	6.4E+01	3.0E+02	1.6E+02	3.7E+02	3.4E+02	4.1E+02	2.5E+02	2.2E+02	1.2E+02	2.1E+02	3.6E+02

Table S9. Standard deviation of the impedance measurements of α-pinene obtained with the $(PAH/GO)_5$-thin-film-based sensor at different frequencies.

Log_{10} Frequency (Hz)	Standard deviation of impedance (Ω)											
	0 ppm	68 ppm	109 ppm	205 ppm	315 ppm	410 ppm	506 ppm	602 ppm	712 ppm	807 ppm	903 ppm	999 ppm
1.0E+06	1.8E+01	2.8E+01	1.3E+01	1.9E+01	3.7E+01	4.5E+01	4.6E+01	3.9E+01	4.1E+01	3.9E+01	4.3E+01	4.5E+01
6.3E+05	2.6E+01	3.3E+01	2.6E+01	2.8E+01	7.1E+01	7.1E+01	7.2E+01	6.9E+01	7.0E+01	6.8E+01	7.1E+01	7.1E+01
4.0E+05	4.0E+01	4.7E+01	4.2E+01	4.3E+01	1.2E+02	1.1E+02	1.2E+02	1.1E+02	1.1E+02	1.1E+02	1.1E+02	1.1E+02
2.5E+05	6.1E+01	7.1E+01	6.7E+01	6.7E+01	1.9E+02	1.8E+02	1.8E+02	1.8E+02	1.8E+02	1.8E+02	1.8E+02	1.8E+02
1.6E+05	9.5E+01	1.1E+02	1.0E+02	1.0E+02	3.0E+02	2.9E+02	2.9E+02	2.9E+02	2.9E+02	2.8E+02	2.9E+02	2.9E+02
1.0E+05	1.6E+02	1.8E+02	1.7E+02	1.7E+02	4.9E+02	4.7E+02	4.7E+02	4.6E+02	4.7E+02	4.6E+02	4.7E+02	4.7E+02
6.3E+04	2.4E+02	2.8E+02	2.7E+02	2.6E+02	7.7E+02	7.4E+02	7.4E+02	7.3E+02	7.4E+02	7.2E+02	7.4E+02	7.4E+02
4.0E+04	3.8E+02	4.4E+02	4.1E+02	4.1E+02	1.2E+03	1.2E+03	1.2E+03	1.2E+03	1.2E+03	1.1E+03	1.2E+03	1.2E+03
2.5E+04	5.8E+02	6.8E+02	6.5E+02	6.4E+02	1.9E+03	1.8E+03	1.8E+03	1.8E+03	1.8E+03	1.8E+03	1.8E+03	1.9E+03
1.6E+04	8.8E+02	1.0E+03	9.9E+02	9.8E+02	3.0E+03	2.9E+03	2.9E+03	2.8E+03	2.9E+03	2.8E+03	2.9E+03	2.9E+03
1.0E+04	1.4E+03	1.6E+03	1.6E+03	1.5E+03	4.8E+03	4.5E+03	4.6E+03	4.5E+03	4.5E+03	4.4E+03	4.6E+03	4.6E+03
6.3E+03	2.1E+03	2.5E+03	2.4E+03	2.4E+03	7.4E+03	7.1E+03	7.2E+03	7.1E+03	7.1E+03	6.9E+03	7.2E+03	7.2E+03
4.0E+03	3.2E+03	4.1E+03	3.6E+03	3.6E+03	1.2E+04	1.1E+04	1.1E+04	1.1E+04	1.1E+04	1.1E+04	1.1E+04	1.1E+04
2.5E+03	4.8E+03	5.9E+03	5.6E+03	5.6E+03	1.8E+04	1.7E+04	1.7E+04	1.7E+04	1.7E+04	1.7E+04	1.7E+04	1.7E+04
1.6E+03	7.1E+03	8.8E+03	8.4E+03	8.4E+03	2.7E+04	2.6E+04	2.6E+04	2.6E+04	2.6E+04	2.6E+04	2.7E+04	2.7E+04
1.0E+03	1.1E+04	1.5E+04	1.1E+04	1.5E+04	4.0E+04	3.9E+04	3.9E+04	3.7E+04	3.6E+04	4.0E+04	4.0E+04	4.0E+04
6.3E+02	2.3E+04	2.2E+04	1.9E+04	2.3E+04	5.6E+04	5.2E+04	5.0E+04	5.3E+04	5.0E+04	6.1E+04	5.8E+04	5.3E+04
4.0E+02	1.9E+04	5.1E+04	3.3E+04	5.2E+04	7.5E+04	7.7E+04	5.8E+04	7.8E+04	7.1E+04	7.5E+04	8.6E+04	7.3E+04
2.5E+02	1.6E+04	8.6E+04	1.9E+04	1.2E+05	5.2E+04	1.1E+05	5.7E+04	3.9E+04	5.9E+04	7.3E+04	1.2E+05	1.7E+05
1.6E+02	2.1E+04	7.8E+04	5.9E+04	7.5E+04	8.8E+04	1.4E+05	1.3E+05	1.0E+05	8.7E+04	8.1E+04	8.9E+04	1.5E+05
1.0E+02	2.8E+05	4.5E+05	7.8E+05	3.0E+05	4.2E+04	6.9E+05	1.3E+05	1.7E+05	5.3E+05	4.2E+05	2.1E+05	3.3E+05
6.3E+01	5.0E+05	1.4E+06	1.9E+06	1.5E+06	1.5E+06	1.8E+05	4.7E+05	8.8E+05	1.5E+05	1.7E+05	8.0E+05	7.8E+05
4.0E+01	3.1E+05	1.9E+06	4.3E+06	1.6E+06	9.5E+04	2.8E+06	1.9E+05	1.2E+06	1.3E+05	3.7E+06	1.4E+06	1.3E+06
2.5E+01	1.1E+06	2.6E+06	2.2E+05	1.6E+06	1.2E+06	9.9E+05	9.2E+04	2.5E+06	1.1E+06	2.0E+06	1.9E+05	1.2E+04
1.6E+01	1.1E+06	4.5E+06	6.2E+06	2.6E+05	3.9E+06	3.4E+06	2.0E+05	3.9E+06	5.1E+06	6.2E+06	5.3E+06	8.6E+05
1.0E+01	6.2E+05	1.1E+06	8.7E+05	1.2E+06	2.3E+06	1.3E+06	6.8E+05	1.2E+06	8.7E+05	7.9E+05	1.0E+06	7.6E+05
6.3E+00	5.0E+05	3.8E+06	6.9E+05	1.3E+06	5.5E+06	6.3E+05	2.8E+06	2.9E+06	3.2E+06	3.1E+06	2.7E+05	7.5E+05
4.0E+00	5.8E+06	1.8E+06	1.5E+06	4.4E+06	1.0E+07	2.0E+05	4.2E+06	1.2E+05	2.0E+06	3.8E+06	4.5E+06	9.3E+05
2.5E+00	2.9E+06	4.3E+06	1.4E+07	2.2E+06	1.8E+06	2.7E+06	3.9E+06	3.7E+06	9.4E+06	3.9E+06	2.8E+04	9.4E+06
1.6E+00	1.1E+07	4.0E+06	2.6E+06	1.3E+06	5.1E+06	2.3E+06	4.7E+06	4.8E+06	8.5E+06	3.5E+05	1.2E+07	1.9E+07
1.0E+00	2.9E+08	5.4E+07	8.4E+06	1.0E+07	2.0E+07	2.9E+05	3.0E+06	3.0E+07	5.5E+06	2.1E+06	8.4E+06	1.6E+06

Table S10. Standard deviation of the impedance measurements of α-pinene obtained with the (PEI/GO)$_5$-thin-film-based sensor at different frequencies.

Log$_{10}$ Frequency (Hz)	Standard deviation of impedance (Ω)											
	0 ppm	68 ppm	109 ppm	205 ppm	315 ppm	410 ppm	506 ppm	602 ppm	712 ppm	807 ppm	903 ppm	999 ppm
1.0E+06	0.0E+00	0.0E+00	0.0E+00	3.9E+01	1.3E+01	2.6E+01	1.7E+01	4.5E+01	3.6E+01	1.6E+01	6.3E+01	6.5E+01
6.3E+05	0.0E+00	0.0E+00	0.0E+00	6.5E+01	2.0E+01	3.5E+01	2.7E+01	7.3E+01	4.8E+01	3.5E+01	1.1E+02	1.1E+02
4.0E+05	0.0E+00	0.0E+00	0.0E+00	1.1E+02	3.1E+01	5.3E+01	4.2E+01	1.2E+02	7.3E+01	5.5E+01	1.9E+02	1.9E+02
2.5E+05	0.0E+00	0.0E+00	0.0E+00	1.7E+02	5.2E+01	8.3E+01	6.5E+01	1.9E+02	1.2E+02	8.7E+01	3.0E+02	3.0E+02
1.6E+05	0.0E+00	0.0E+00	0.0E+00	2.8E+02	8.2E+01	1.3E+02	1.0E+02	3.1E+02	1.9E+02	1.4E+02	4.9E+02	4.8E+02
1.0E+05	0.0E+00	0.0E+00	0.0E+00	4.6E+02	1.4E+02	2.1E+02	1.6E+02	4.9E+02	3.0E+02	2.3E+02	7.8E+02	7.8E+02
6.3E+04	0.0E+00	0.0E+00	0.0E+00	7.3E+02	2.2E+02	3.2E+02	2.5E+02	7.9E+02	4.8E+02	3.7E+02	1.2E+03	1.2E+03
4.0E+04	0.0E+00	0.0E+00	0.0E+00	1.2E+03	3.5E+02	5.0E+02	4.0E+02	1.2E+03	7.7E+02	5.8E+02	2.0E+03	2.0E+03
2.5E+04	0.0E+00	0.0E+00	0.0E+00	1.8E+03	5.6E+02	7.9E+02	6.3E+02	2.0E+03	1.2E+03	9.3E+02	3.1E+03	3.1E+03
1.6E+04	0.0E+00	0.0E+00	0.0E+00	2.9E+03	8.8E+02	1.3E+03	9.9E+02	3.1E+03	1.9E+03	1.5E+03	4.9E+03	4.9E+03
1.0E+04	0.0E+00	0.0E+00	0.0E+00	4.6E+03	1.4E+03	2.0E+03	1.6E+03	4.9E+03	3.1E+03	2.3E+03	7.8E+03	7.7E+03
6.3E+03	0.0E+00	0.0E+00	0.0E+00	7.2E+03	2.2E+03	3.1E+03	2.5E+03	7.8E+03	4.9E+03	3.7E+03	1.2E+04	1.2E+04
4.0E+03	0.0E+00	0.0E+00	0.0E+00	1.1E+04	3.5E+03	4.7E+03	4.0E+03	1.2E+04	8.3E+03	6.0E+03	1.9E+04	1.9E+04
2.5E+03	0.0E+00	0.0E+00	0.0E+00	1.8E+04	5.3E+03	7.1E+03	6.2E+03	1.9E+04	1.3E+04	8.9E+03	3.0E+04	3.0E+04
1.6E+03	0.0E+00	0.0E+00	0.0E+00	2.8E+04	8.3E+03	9.4E+03	9.9E+03	3.0E+04	2.4E+04	1.4E+04	4.7E+04	4.7E+04
1.0E+03	0.0E+00	0.0E+00	0.0E+00	4.3E+04	1.4E+04	8.3E+03	1.5E+04	4.9E+04	3.7E+04	2.2E+04	7.5E+04	7.4E+04
6.3E+02	0.0E+00	0.0E+00	0.0E+00	7.0E+04	2.1E+04	1.4E+04	3.2E+04	7.6E+04	6.1E+04	2.6E+04	1.2E+05	1.1E+05
4.0E+02	0.0E+00	0.0E+00	0.0E+00	1.1E+05	2.9E+03	7.4E+03	3.4E+04	9.1E+04	8.3E+04	2.9E+04	1.9E+05	2.0E+05
2.5E+02	0.0E+00	0.0E+00	0.0E+00	1.4E+05	2.0E+04	1.9E+04	5.9E+04	1.3E+05	2.2E+05	6.6E+04	2.9E+05	2.6E+05
1.6E+02	0.0E+00	0.0E+00	0.0E+00	2.3E+05	6.7E+04	1.0E+05	1.5E+05	3.0E+05	2.5E+05	1.5E+05	3.5E+05	4.5E+05
1.0E+02	0.0E+00	0.0E+00	0.0E+00	1.7E+04	2.2E+05	5.0E+04	7.2E+05	9.0E+05	1.7E+04	5.0E+05	4.8E+05	8.4E+05
6.3E+01	0.0E+00	0.0E+00	0.0E+00	1.0E+05	3.5E+05	6.2E+05	4.0E+05	5.3E+05	3.8E+05	1.2E+06	1.3E+06	1.9E+05
4.0E+01	0.0E+00	0.0E+00	0.0E+00	2.7E+06	2.7E+06	4.2E+06	9.8E+05	2.5E+06	1.3E+06	7.7E+05	1.2E+06	2.1E+06
2.5E+01	0.0E+00	0.0E+00	0.0E+00	2.2E+06	1.1E+06	7.5E+05	2.1E+06	2.0E+06	1.7E+05	2.2E+06	8.0E+05	1.3E+06
1.6E+01	0.0E+00	0.0E+00	0.0E+00	3.3E+05	3.8E+06	0.0E+00	1.2E+06	5.2E+06	2.7E+06	4.0E+06	5.0E+06	3.8E+06
1.0E+01	0.0E+00	0.0E+00	0.0E+00	3.7E+06	1.0E+06	1.2E+06	6.7E+05	1.0E+06	3.7E+06	1.0E+06	1.5E+06	5.3E+06
6.3E+00	0.0E+00	0.0E+00	0.0E+00	2.0E+06	6.7E+05	2.5E+06	1.3E+06	5.0E+05	4.8E+06	9.5E+06	1.0E+06	1.2E+06
4.0E+00	0.0E+00	0.0E+00	0.0E+00	1.0E+06	6.2E+06	7.2E+06	5.0E+05	6.3E+06	9.2E+06	5.0E+06	3.3E+05	3.3E+07
2.5E+00	0.0E+00	0.0E+00	0.0E+00	1.4E+07	1.2E+07	1.7E+07	3.3E+05	6.7E+06	6.7E+06	5.0E+05	1.8E+07	1.4E+07
1.6E+00	0.0E+00	0.0E+00	0.0E+00	6.7E+06	1.7E+06	1.7E+06	1.7E+06	1.2E+07	2.5E+07	6.7E+06	6.7E+07	4.4E+06
1.0E+00	0.0E+00	0.0E+00	0.0E+00	5.3E+07	3.7E+07	2.5E+07	7.2E+07	7.2E+07	3.7E+07	1.8E+07	7.2E+07	2.6E+07

Table S11. Standard deviation of the impedance measurements of α-pinene obtained with the (PAH/MWCNT)$_5$-thin-film-based sensor at different frequencies.

Log$_{10}$ Frequency (Hz)	Standard deviation of impedance (Ω)											
	0 ppm	68 ppm	109 ppm	205 ppm	315 ppm	410 ppm	506 ppm	602 ppm	712 ppm	807 ppm	903 ppm	999 ppm
1.0E+06	8.8E+01	2.4E+02	3.5E+02	4.0E+02	3.5E+02	3.4E+02	2.7E+02	1.3E+02	3.4E+01	1.0E+02	2.2E+02	4.3E+01
6.3E+05	8.0E+01	4.2E+02	4.0E+02	3.6E+02	2.4E+02	3.8E+02	2.0E+02	2.1E+02	1.5E+02	1.5E+02	4.2E+02	7.6E+01
4.0E+05	1.4E+02	1.2E+02	2.0E+02	1.7E+02	3.1E+02	1.2E+02	4.1E+02	4.2E+02	4.0E+02	3.5E+02	4.1E+02	4.3E+02
2.5E+05	3.9E+01	3.4E+01	3.0E+02	3.7E+02	1.9E+02	3.7E+01	1.8E+02	1.9E+01	4.6E+02	2.9E+02	2.2E+02	3.8E+02
1.6E+05	5.5E+01	3.4E+02	2.3E+01	2.6E+02	4.4E+02	2.5E+02	9.7E+01	5.8E+01	3.3E+02	1.9E+02	3.2E+02	2.2E+02
1.0E+05	3.1E+02	1.1E+02	2.5E+02	2.7E+01	3.0E+02	4.1E+02	9.6E+01	1.7E+02	1.3E+02	1.2E+02	2.7E+02	2.0E+02
6.3E+04	4.4E+02	1.3E+02	2.4E+02	1.0E+02	5.7E+01	1.2E+02	4.2E+02	2.2E+02	3.5E+02	1.8E+01	4.1E+02	4.2E+02
4.0E+04	2.9E+02	2.3E+02	1.1E+02	4.7E+01	3.9E+02	2.7E+02	4.6E+02	4.1E+01	3.5E+02	2.8E+02	3.9E+02	2.9E+02
2.5E+04	2.2E+02	4.6E+02	4.7E+02	3.0E+02	2.0E+02	3.2E+02	2.8E+01	3.8E+01	4.2E+02	4.2E+02	2.9E+02	3.4E+02
1.6E+04	2.2E+02	4.6E+02	1.3E+02	4.1E+02	3.9E+02	4.0E+02	2.7E+02	3.7E+02	5.8E+01	1.6E+02	1.8E+02	1.4E+02
1.0E+04	3.0E+02	1.7E+02	1.6E+02	6.1E+01	4.2E+02	2.0E+02	3.8E+01	3.2E+02	3.4E+02	1.6E+02	3.2E+01	2.2E+02
6.3E+03	2.1E+02	2.1E+02	4.7E+02	2.6E+02	2.3E+02	3.8E+01	4.5E+02	6.4E+01	2.8E+02	1.7E+02	3.9E+02	3.3E+02
4.0E+03	3.9E+02	3.2E+02	4.6E+02	3.8E+02	2.7E+02	1.5E+02	9.5E+01	3.4E+02	4.8E+02	3.4E+02	6.7E+01	9.2E+01
2.5E+03	5.8E+01	3.3E+02	4.8E+02	3.5E+02	1.9E+02	2.8E+02	2.5E+02	4.3E+02	2.6E+02	2.8E+02	2.3E+02	3.2E+01
1.6E+03	2.0E+02	4.1E+02	4.8E+02	1.9E+02	4.0E+02	2.5E+02	3.2E+02	1.5E+02	1.0E+02	2.7E+02	3.5E+02	3.3E+02

1.0E+03	4.3E+02	3.0E+02	2.6E+02	1.3E+02	1.9E+02	3.2E+02	3.1E+02	2.0E+01	3.7E+02	3.8E+02	4.0E+02	2.4E+02
6.3E+02	9.9E+01	1.7E+02	4.3E+02	3.4E+02	4.0E+02	4.7E+02	9.0E+01	3.0E+02	1.7E+02	2.4E+01	3.7E+02	1.4E+02
4.0E+02	4.0E+02	2.5E+02	3.3E+02	4.5E+02	6.1E+01	4.4E+02	2.4E+02	8.8E+01	5.4E+01	4.7E+02	3.0E+02	2.9E+02
2.5E+02	7.0E+01	2.4E+02	3.0E+02	7.3E+01	1.8E+02	6.8E+01	4.5E+02	4.8E+02	2.7E+02	2.9E+02	5.4E+01	2.5E+02
1.6E+02	1.1E+02	1.2E+02	4.3E+02	8.1E+01	3.5E+02	3.2E+02	4.2E+02	4.0E+02	1.6E+02	1.1E+02	2.2E+02	1.4E+02
1.0E+02	1.3E+02	5.0E+01	2.9E+02	8.3E+01	3.3E+02	2.4E+02	3.9E+02	4.4E+02	7.4E+01	8.6E+01	1.8E+02	9.3E+01
6.3E+01	4.1E+02	3.1E+02	1.9E+02	2.3E+02	3.5E+02	1.3E+02	5.8E+01	2.3E+02	4.0E+02	1.9E+02	3.6E+02	4.6E+02
4.0E+01	5.0E+01	8.9E+01	2.3E+02	3.6E+02	2.8E+02	4.3E+02	3.1E+02	4.0E+01	2.6E+02	3.4E+02	2.7E+02	1.7E+02
2.5E+01	3.2E+02	2.8E+02	3.2E+02	1.8E+02	1.7E+02	1.3E+02	1.5E+02	4.4E+01	1.2E+02	4.6E+02	1.5E+02	2.0E+02
1.6E+01	2.7E+02	4.7E+01	3.2E+02	3.3E+02	3.2E+02	1.7E+02	1.2E+02	1.6E+02	2.6E+02	3.2E+02	2.3E+02	4.4E+02
1.0E+01	4.1E+02	4.4E+02	3.4E+02	3.2E+02	1.6E+02	4.2E+01	1.3E+02	5.9E+01	2.7E+02	3.2E+02	1.8E+02	4.0E+02
6.3E+00	1.1E+02	1.4E+02	4.5E+02	2.4E+01	3.4E+02	2.4E+02	1.6E+02	3.6E+02	8.5E+01	1.3E+02	3.8E+02	4.3E+02
4.0E+00	9.7E+01	2.8E+02	1.2E+02	2.7E+02	3.8E+01	4.6E+02	4.7E+02	9.1E+01	2.5E+02	2.6E+02	4.7E+02	3.4E+02
2.5E+00	4.2E+02	4.7E+02	1.5E+02	7.7E+01	1.4E+02	4.3E+02	2.8E+02	2.9E+02	4.7E+02	7.7E+01	3.4E+02	3.1E+02
1.6E+00	1.2E+02	4.6E+02	2.7E+02	3.1E+02	1.2E+02	2.6E+02	2.9E+02	4.7E+02	2.0E+01	6.0E+01	4.1E+02	4.5E+02
1.0E+00	2.9E+01	3.4E+02	1.3E+02	4.3E+02	2.5E+02	1.9E+02	8.8E+01	2.5E+02	3.9E+02	1.6E+02	5.0E+01	1.4E+02

Table S12. Standard deviation of the impedance measurements of α-pinene obtained with the (PAH/MWCNT-COOH)$_5$-thin-film-based sensor at different frequencies.

**Log_{10} Frequency (Hz)**	**Standard deviation of impedance (Ω)**											
	**0 ppm**	**68 ppm**	**109 ppm**	**205 ppm**	**315 ppm**	**410 ppm**	**506 ppm**	**602 ppm**	**712 ppm**	**807 ppm**	**903 ppm**	**999 ppm**
1.0E+06	4.2E+02	3.9E+02	4.6E+02	1.3E+02	1.4E+02	2.3E+02	3.5E+02	1.2E+02	4.0E+02	4.1E+02	2.1E+02	2.0E+02
6.3E+05	2.1E+02	2.3E+02	2.5E+02	1.8E+02	6.9E+01	2.7E+02	2.6E+02	2.5E+02	2.7E+02	6.8E+01	2.1E+02	3.5E+02
4.0E+05	1.1E+02	7.8E+01	4.7E+02	4.6E+01	1.1E+02	4.5E+02	2.2E+02	4.3E+02	4.2E+02	3.6E+01	1.7E+02	4.4E+02
2.5E+05	2.1E+02	3.8E+02	3.6E+02	2.1E+02	2.3E+02	1.8E+02	3.1E+02	3.2E+02	4.2E+02	7.3E+01	4.0E+01	2.6E+02
1.6E+05	1.1E+02	2.6E+02	4.7E+02	1.9E+02	2.0E+02	4.2E+02	2.4E+02	1.0E+02	6.0E+01	3.8E+02	1.7E+02	4.0E+01
1.0E+05	2.8E+02	1.1E+02	1.1E+02	7.2E+01	4.1E+01	4.1E+02	4.0E+02	4.3E+02	1.3E+02	4.0E+02	3.0E+02	3.4E+02
6.3E+04	1.4E+02	1.2E+02	1.4E+02	2.4E+02	1.3E+02	9.9E+01	3.7E+02	4.6E+02	3.5E+02	3.4E+02	6.2E+01	1.7E+02
4.0E+04	2.0E+02	1.5E+02	8.4E+01	7.6E+01	3.2E+01	1.7E+02	1.0E+02	1.9E+02	1.7E+02	9.4E+01	2.8E+02	3.0E+02
2.5E+04	2.0E+02	2.8E+01	2.9E+02	3.5E+02	1.2E+02	1.9E+02	2.2E+02	3.2E+02	3.4E+02	2.5E+02	3.6E+02	2.2E+02
1.6E+04	1.3E+02	2.1E+02	1.0E+02	2.3E+02	3.2E+02	1.4E+02	4.4E+02	3.9E+01	2.1E+02	3.2E+02	3.4E+02	2.7E+02
1.0E+04	2.3E+02	3.3E+02	3.4E+02	1.9E+02	4.0E+02	8.6E+01	1.4E+02	2.1E+02	3.6E+01	2.8E+01	5.3E+01	4.2E+02
6.3E+03	2.2E+02	9.2E+01	2.2E+01	2.0E+02	3.2E+02	4.0E+02	2.6E+02	7.0E+01	6.7E+01	3.6E+02	4.2E+02	2.8E+02
4.0E+03	1.2E+02	2.7E+02	3.3E+02	4.2E+02	4.7E+02	3.3E+02	2.5E+02	6.6E+01	1.1E+02	3.4E+02	4.1E+02	9.0E+01
2.5E+03	9.1E+01	4.0E+02	4.7E+02	4.1E+02	1.1E+02	4.7E+02	1.0E+02	3.1E+02	2.0E+02	1.5E+02	3.8E+02	4.5E+02
1.6E+03	4.4E+02	1.5E+02	4.6E+02	4.2E+02	1.1E+02	4.3E+02	3.0E+02	1.7E+02	1.4E+02	3.3E+02	2.5E+02	4.4E+01
1.0E+03	9.9E+01	1.7E+02	1.3E+02	3.7E+02	3.4E+02	8.7E+01	1.7E+02	3.0E+02	4.3E+02	9.0E+01	3.7E+02	2.0E+02
6.3E+02	5.9E+01	2.9E+02	1.4E+02	3.9E+02	2.8E+02	4.4E+02	4.0E+02	3.3E+02	1.3E+02	4.6E+02	1.3E+02	1.5E+02
4.0E+02	4.5E+02	1.6E+02	3.5E+02	2.8E+02	2.1E+02	2.7E+02	1.5E+02	2.1E+02	2.5E+02	2.5E+02	1.9E+02	3.2E+02
2.5E+02	6.6E+01	1.9E+02	1.4E+02	3.9E+02	1.1E+02	5.1E+01	3.0E+01	1.3E+02	3.8E+02	1.4E+02	8.1E+01	2.4E+02
1.6E+02	1.2E+02	1.0E+02	1.1E+02	1.8E+02	3.0E+02	3.6E+02	2.9E+02	4.5E+02	2.0E+02	3.7E+02	2.3E+02	1.7E+02
1.0E+02	2.5E+02	2.6E+01	7.2E+01	4.7E+02	3.9E+02	3.6E+02	4.6E+02	6.6E+01	3.4E+02	1.7E+02	4.2E+02	1.4E+02
6.3E+01	2.0E+02	1.1E+02	2.0E+01	2.3E+02	4.4E+02	1.4E+02	4.6E+02	2.7E+02	2.9E+02	2.5E+02	2.7E+02	3.8E+02
4.0E+01	8.0E+01	2.4E+02	6.5E+01	1.8E+02	2.2E+02	2.8E+02	2.7E+02	3.8E+02	7.4E+01	1.3E+02	2.0E+01	1.7E+02
2.5E+01	1.0E+02	1.6E+02	2.6E+02	4.6E+02	3.2E+02	2.0E+02	4.1E+01	5.1E+01	6.3E+01	1.7E+02	2.6E+02	4.2E+02
1.6E+01	2.1E+02	6.8E+01	4.7E+02	4.0E+02	4.7E+01	1.7E+02	2.6E+02	8.0E+01	4.2E+02	4.6E+01	4.9E+01	5.3E+01
1.0E+01	1.5E+02	3.4E+02	3.9E+02	8.1E+01	2.6E+02	3.8E+01	2.9E+02	2.7E+02	2.5E+02	1.4E+02	1.0E+02	2.1E+02
6.3E+00	3.3E+02	2.1E+02	7.2E+01	4.3E+02	1.0E+02	4.2E+02	3.6E+02	2.6E+02	4.1E+02	3.9E+02	2.0E+01	2.8E+02
4.0E+00	1.2E+02	4.2E+02	4.2E+01	1.5E+02	3.7E+02	3.7E+02	3.6E+02	2.8E+02	4.5E+02	2.4E+02	2.5E+02	5.9E+01
2.5E+00	4.5E+02	2.1E+01	1.3E+02	3.8E+02	4.1E+02	2.0E+02	4.1E+02	2.9E+02	2.0E+02	3.4E+02	1.6E+02	3.9E+02
1.6E+00	2.8E+02	3.6E+02	3.0E+02	3.4E+02	5.5E+01	4.4E+02	1.2E+02	3.7E+02	2.2E+02	1.8E+02	3.3E+02	3.1E+02
1.0E+00	1.6E+02	4.3E+02	1.3E+02	2.0E+02	3.6E+02	1.9E+02	1.1E+02	1.5E+02	2.6E+02	3.1E+02	7.1E+01	3.0E+02

Table S13. Standard deviation of the impedance measurements of α-pinene obtained with the ZnO (50% O_2)-thin-film-based sensor at different frequencies.

Log_{10} Frequency (Hz)	Standard deviation of impedance (Ω)											
	0 ppm	68 ppm	109 ppm	205 ppm	315 ppm	410 ppm	506 ppm	602 ppm	712 ppm	807 ppm	903 ppm	999 ppm
1.0E+06	1.2E+01	1.6E+01	1.1E+01	1.3E+01	1.1E+01	3.1E+01	2.0E+01	1.2E+01	3.6E+01	3.7E+01	3.7E+01	3.4E+01
6.3E+05	2.0E+01	2.3E+01	2.1E+01	2.1E+01	2.1E+01	4.5E+01	2.7E+01	2.0E+01	6.2E+01	6.2E+01	6.2E+01	6.0E+01
4.0E+05	3.3E+01	3.6E+01	3.5E+01	3.6E+01	3.4E+01	6.0E+01	3.8E+01	3.2E+01	1.0E+02	1.0E+02	1.0E+02	9.9E+01
2.5E+05	5.2E+01	6.0E+01	5.7E+01	5.8E+01	5.6E+01	7.7E+01	5.8E+01	5.1E+01	1.6E+02	1.6E+02	1.6E+02	1.6E+02
1.6E+05	8.2E+01	9.6E+01	9.2E+01	9.4E+01	8.9E+01	1.1E+02	9.2E+01	8.1E+01	2.6E+02	2.6E+02	2.6E+02	2.6E+02
1.0E+05	1.4E+02	1.6E+02	1.5E+02	1.6E+02	1.5E+02	1.6E+02	1.5E+02	1.3E+02	4.2E+02	4.2E+02	4.2E+02	4.1E+02
6.3E+04	2.2E+02	2.6E+02	2.4E+02	2.5E+02	2.3E+02	2.4E+02	2.3E+02	2.1E+02	6.6E+02	6.6E+02	6.7E+02	6.5E+02
4.0E+04	3.5E+02	4.0E+02	3.9E+02	3.9E+02	3.7E+02	3.7E+02	3.7E+02	3.3E+02	1.0E+03	1.0E+03	1.1E+03	1.0E+03
2.5E+04	5.4E+02	6.4E+02	6.1E+02	6.2E+02	5.8E+02	5.8E+02	5.7E+02	5.2E+02	1.7E+03	1.6E+03	1.7E+03	1.6E+03
1.6E+04	8.4E+02	1.0E+03	9.5E+02	9.7E+02	9.1E+02	9.0E+02	9.0E+02	8.0E+02	2.6E+03	2.6E+03	2.6E+03	2.6E+03
1.0E+04	1.4E+03	1.6E+03	1.5E+03	1.5E+03	1.5E+03	1.4E+03	1.4E+03	1.3E+03	4.1E+03	4.0E+03	4.2E+03	4.1E+03
6.3E+03	2.1E+03	2.4E+03	2.4E+03	2.4E+03	2.3E+03	2.2E+03	2.2E+03	2.0E+03	6.5E+03	5.9E+03	6.5E+03	6.4E+03
4.0E+03	3.3E+03	3.5E+03	3.7E+03	3.8E+03	3.4E+03	3.5E+03	3.4E+03	3.4E+03	9.8E+03	8.2E+03	9.8E+03	1.0E+04
2.5E+03	5.2E+03	5.9E+03	5.8E+03	5.8E+03	5.4E+03	5.3E+03	5.4E+03	4.9E+03	1.5E+04	1.1E+04	1.5E+04	1.6E+04
1.6E+03	7.7E+03	9.0E+03	8.7E+03	8.9E+03	8.5E+03	8.3E+03	8.3E+03	7.5E+03	2.3E+04	1.3E+04	2.3E+04	2.4E+04
1.0E+03	1.2E+04	1.6E+04	1.4E+04	1.5E+04	1.4E+04	1.3E+04	1.3E+04	1.1E+04	3.3E+04	1.5E+04	3.1E+04	3.8E+04
6.3E+02	1.5E+04	2.0E+04	2.2E+04	2.1E+04	2.1E+04	1.4E+04	2.3E+04	1.3E+04	3.3E+04	2.2E+04	4.6E+04	5.9E+04
4.0E+02	3.7E+04	8.0E+03	4.4E+04	3.4E+04	3.7E+04	2.8E+04	2.9E+04	2.5E+04	7.1E+04	4.1E+04	5.3E+04	9.8E+04
2.5E+02	2.7E+04	9.2E+04	5.4E+03	1.1E+05	6.1E+04	1.3E+05	3.7E+04	5.2E+04	4.6E+04	3.2E+04	3.3E+04	9.6E+04
1.6E+02	6.9E+04	6.4E+04	9.7E+04	9.5E+04	4.2E+04	1.4E+04	1.1E+05	2.7E+04	9.9E+04	7.4E+04	8.5E+04	2.2E+05
1.0E+02	7.2E+04	2.5E+05	1.4E+05	3.0E+05	3.2E+05	1.3E+05	9.3E+05	1.1E+06	3.8E+05	5.0E+05	1.2E+05	4.1E+05
6.3E+01	1.4E+06	1.4E+06	4.8E+05	5.4E+05	2.4E+05	2.1E+06	1.1E+06	1.1E+05	1.0E+06	9.5E+05	1.7E+06	6.6E+05
4.0E+01	2.4E+05	1.2E+06	1.3E+06	3.0E+06	7.9E+05	4.5E+05	1.3E+06	1.0E+06	2.5E+06	1.1E+06	2.2E+06	9.3E+05
2.5E+01	4.0E+05	2.4E+06	2.2E+05	1.2E+06	1.8E+06	1.1E+06	1.9E+06	4.5E+05	1.2E+06	1.5E+06	9.5E+05	2.3E+06
1.6E+01	3.6E+06	4.6E+06	7.1E+06	2.2E+05	1.2E+05	6.5E+05	1.0E+06	1.2E+06	2.0E+06	1.3E+05	2.1E+06	4.7E+06
1.0E+01	6.9E+04	1.3E+06	1.6E+06	1.2E+06	1.3E+06	1.3E+06	1.5E+06	1.3E+06	1.1E+06	1.4E+06	9.3E+05	6.0E+05
6.3E+00	7.1E+05	8.3E+05	1.0E+06	1.2E+06	1.6E+06	2.3E+05	1.5E+06	4.4E+05	3.2E+06	3.5E+06	1.5E+06	2.1E+06
4.0E+00	1.2E+06	2.6E+05	5.5E+06	4.4E+07	2.7E+07	3.8E+06	3.0E+06	2.7E+06	6.0E+06	6.5E+06	5.3E+05	7.4E+05
2.5E+00	1.6E+07	1.1E+07	1.0E+07	5.3E+05	2.2E+07	6.6E+06	4.2E+07	9.8E+06	2.8E+07	7.1E+06	1.2E+07	1.9E+07
1.6E+00	8.4E+07	3.0E+07	2.8E+07	1.8E+07	2.2E+07	1.7E+07	4.8E+07	2.1E+07	2.1E+07	1.4E+07	3.2E+07	1.8E+07
1.0E+00	7.4E+07	1.6E+08	1.7E+08	3.2E+07	1.2E+08	7.5E+07	3.7E+07	6.7E+07	1.1E+09	4.9E+07	2.8E+07	4.9E+07

Table S14. Standard deviation of the impedance measurements of α-pinene obtained with the ZnO (100% O_2)-thin-film-based sensor at different frequencies.

Log_{10} Frequency (Hz)	Standard deviation of impedance (Ω)											
	0 ppm	68 ppm	109 ppm	205 ppm	315 ppm	410 ppm	506 ppm	602 ppm	712 ppm	807 ppm	903 ppm	999 ppm
1.0E+06	0.0E+00	8.0E+00	8.3E+00	6.0E+00	5.0E+00	4.0E+00	8.1E-01	3.4E+00	4.7E+00	5.0E+00	7.1E+00	5.7E+00
6.3E+05	7.0E+00	9.8E+00	1.2E+01	1.3E+01	8.8E+00	7.4E+00	7.3E+00	7.2E+00	7.3E+00	7.8E+00	8.3E+00	8.6E+00
4.0E+05	1.3E+01	1.4E+01	2.0E+01	3.0E+01	1.4E+01	1.3E+01	1.4E+01	1.3E+01	1.2E+01	1.3E+01	1.3E+01	1.4E+01
2.5E+05	2.1E+01	2.3E+01	3.2E+01	7.0E+01	2.3E+01	2.1E+01	2.4E+01	2.0E+01	1.9E+01	2.1E+01	2.0E+01	2.2E+01
1.6E+05	3.2E+01	3.4E+01	5.1E+01	1.5E+02	3.7E+01	3.3E+01	4.0E+01	3.1E+01	3.1E+01	3.2E+01	3.2E+01	3.5E+01
1.0E+05	5.7E+01	5.7E+01	8.1E+01	3.0E+02	6.3E+01	5.8E+01	6.7E+01	5.3E+01	5.1E+01	5.4E+01	5.5E+01	5.7E+01
6.3E+04	8.8E+01	7.7E+01	1.1E+02	5.2E+02	1.0E+02	9.3E+01	1.1E+02	8.4E+01	8.3E+01	8.8E+01	8.6E+01	9.3E+01
4.0E+04	1.4E+02	1.1E+02	1.3E+02	8.5E+02	1.6E+02	1.5E+02	1.7E+02	1.3E+02	1.4E+02	1.4E+02	1.4E+02	1.5E+02
2.5E+04	2.1E+02	4.2E+01	5.6E+01	1.4E+03	2.5E+02	2.3E+02	2.7E+02	2.2E+02	2.1E+02	2.3E+02	2.2E+02	2.3E+02
1.6E+04	3.0E+02	3.3E+02	1.7E+02	2.2E+03	3.9E+02	3.5E+02	4.3E+02	3.3E+02	3.2E+02	3.4E+02	3.4E+02	3.7E+02
1.0E+04	5.2E+02	8.4E+02	1.3E+02	3.5E+03	6.5E+02	5.8E+02	7.3E+02	5.5E+02	5.2E+02	5.5E+02	5.6E+02	5.9E+02
6.3E+03	8.0E+02	1.5E+03	3.9E+02	5.5E+03	1.0E+03	9.5E+02	1.3E+03	8.7E+02	8.1E+02	8.6E+02	9.1E+02	9.3E+02
4.0E+03	1.2E+03	2.0E+03	9.2E+02	8.6E+03	1.3E+03	1.4E+03	2.3E+03	1.4E+03	1.1E+03	1.2E+03	1.2E+03	1.3E+03
2.5E+03	1.7E+03	4.9E+03	1.3E+03	1.3E+04	2.5E+03	2.2E+03	4.7E+03	2.1E+03	2.0E+03	2.2E+03	2.1E+03	2.3E+03
1.6E+03	2.5E+03	8.2E+03	4.9E+03	2.0E+04	3.6E+03	3.5E+03	9.9E+03	3.3E+03	3.2E+03	3.5E+03	3.5E+03	3.7E+03

1.0E+03	4.0E+03	1.5E+04	1.3E+03	2.9E+04	5.8E+03	4.2E+03	2.1E+04	6.1E+03	5.0E+03	5.9E+03	5.1E+03	4.8E+03
6.3E+02	4.8E+03	2.0E+04	6.5E+03	4.0E+04	1.1E+04	6.9E+03	4.6E+04	2.0E+04	1.1E+04	1.1E+03	9.3E+03	1.6E+04
4.0E+02	1.9E+04	4.5E+04	2.9E+04	7.5E+04	6.1E+03	1.3E+04	7.0E+04	4.4E+04	6.8E+03	2.6E+04	2.0E+04	5.4E+03
2.5E+02	3.2E+04	1.2E+03	4.3E+04	6.5E+04	6.9E+03	4.5E+04	1.8E+03	8.8E+04	9.5E+04	7.2E+04	4.2E+04	4.4E+04
1.6E+02	6.5E+03	1.3E+05	8.9E+04	1.5E+05	3.5E+04	1.3E+04	1.7E+05	1.1E+05	1.5E+04	4.6E+04	3.0E+04	6.1E+04
1.0E+02	9.5E+05	4.0E+05	8.2E+04	9.1E+05	2.2E+05	2.6E+05	2.3E+05	8.8E+05	3.7E+05	2.8E+05	2.2E+05	2.5E+05
6.3E+01	1.4E+06	9.5E+05	5.9E+05	1.0E+06	1.6E+06	4.8E+05	1.1E+06	1.2E+06	1.1E+06	1.4E+06	3.0E+05	5.5E+05
4.0E+01	1.3E+06	1.5E+06	1.0E+06	6.9E+05	1.4E+06	1.3E+06	5.3E+06	3.8E+06	1.4E+06	4.0E+06	3.7E+06	2.4E+06
2.5E+01	7.3E+05	1.6E+06	2.4E+05	1.8E+06	3.7E+06	4.0E+06	1.5E+06	3.4E+05	4.8E+05	4.8E+03	3.5E+05	1.0E+06
1.6E+01	3.0E+06	2.2E+04	3.2E+06	1.8E+05	3.1E+06	2.6E+06	3.8E+05	6.4E+06	2.0E+06	2.5E+06	5.0E+05	1.3E+06
1.0E+01	7.5E+06	5.7E+06	6.8E+06	5.9E+06	4.7E+06	4.8E+06	4.8E+05	3.4E+06	1.9E+06	3.7E+06	1.2E+06	7.1E+05
6.3E+00	2.5E+07	1.9E+07	1.9E+07	1.9E+07	1.6E+07	1.3E+07	1.8E+06	6.3E+06	5.8E+06	7.9E+06	2.1E+06	1.6E+07
4.0E+00	5.1E+07	5.2E+07	4.0E+07	3.5E+07	3.4E+07	2.6E+07	1.6E+06	8.9E+06	1.1E+07	8.4E+06	1.0E+07	8.3E+06
2.5E+00	9.8E+07	8.2E+07	7.3E+07	6.0E+07	5.2E+07	4.6E+07	7.6E+06	1.8E+07	2.0E+07	1.8E+07	1.5E+07	1.4E+07
1.6E+00	1.4E+08	1.1E+08	1.0E+08	8.2E+07	7.3E+07	6.2E+07	8.1E+06	2.2E+07	2.5E+07	2.5E+07	1.4E+07	1.9E+07
1.0E+00	1.5E+08	1.5E+08	1.2E+08	9.3E+07	8.3E+07	7.3E+07	1.1E+07	2.6E+07	3.1E+07	2.9E+07	2.2E+07	2.3E+07

Table S15. Standard deviation of the impedance measurements of α-pinene obtained with the TiO_2 (50% O_2)-thin-film-based sensor at different frequencies.

Log_{10} Frequency (Hz)	Standard deviation of impedance (Ω)											
	0 ppm	68 ppm	109 ppm	205 ppm	315 ppm	410 ppm	506 ppm	602 ppm	712 ppm	807 ppm	903 ppm	999 ppm
1.0E+06	1.8E+00	5.9E+01	1.2E+01	2.3E+01	6.7E+00	5.2E+00	1.3E+01	1.1E+02	1.8E+00	5.9E+00	8.0E+01	1.9E+01
6.3E+05	2.2E+00	8.0E-02	3.2E+01	4.0E+01	7.4E+00	8.3E+00	1.3E+01	1.1E+02	5.4E+00	6.2E+00	3.9E+01	3.7E+01
4.0E+05	5.0E+00	1.2E-01	5.3E+01	6.5E+01	1.1E+01	1.3E+01	1.6E+01	8.2E+01	1.0E+01	8.0E+00	6.4E+01	6.3E+01
2.5E+05	7.7E+00	3.2E-01	7.9E+01	1.1E+02	2.0E+01	2.2E+01	2.2E+01	1.4E+01	1.7E+01	1.2E+01	1.0E+02	1.0E+02
1.6E+05	1.3E+01	5.7E-01	1.2E+02	1.7E+02	3.2E+01	3.5E+01	3.3E+01	7.9E+01	2.7E+01	1.9E+01	1.6E+02	1.7E+02
1.0E+05	2.0E+01	1.1E+00	1.9E+02	2.7E+02	5.4E+01	5.9E+01	5.3E+01	2.0E+02	4.5E+01	3.3E+01	2.6E+02	2.6E+02
6.3E+04	3.3E+01	1.3E+00	2.9E+02	4.3E+02	8.6E+01	9.3E+01	8.3E+01	3.7E+02	7.5E+01	5.4E+01	4.1E+02	4.2E+02
4.0E+04	5.2E+01	1.2E+01	4.5E+02	6.7E+02	1.4E+02	1.5E+02	1.3E+02	6.1E+02	1.2E+02	8.9E+01	6.4E+02	6.5E+02
2.5E+04	8.0E+01	4.1E+00	7.0E+02	1.1E+03	2.2E+02	2.4E+02	2.0E+02	9.1E+02	1.9E+02	1.4E+02	1.0E+03	1.0E+03
1.6E+04	1.3E+02	6.0E+00	1.1E+03	1.7E+03	3.5E+02	3.7E+02	3.3E+02	1.2E+03	3.0E+02	2.2E+02	1.6E+03	1.5E+03
1.0E+04	1.9E+02	2.2E+01	1.7E+03	2.6E+03	5.5E+02	6.0E+02	5.2E+02	1.3E+03	4.8E+02	3.7E+02	2.5E+03	2.1E+03
6.3E+03	3.2E+02	3.5E+01	2.9E+03	4.1E+03	8.8E+02	9.1E+02	7.6E+02	1.0E+03	7.7E+02	5.7E+02	5.9E+03	2.5E+03
4.0E+03	5.2E+02	3.3E+02	6.0E+03	6.7E+03	1.3E+03	1.6E+03	1.5E+03	5.7E+02	1.1E+03	1.0E+03	2.0E+04	2.3E+03
2.5E+03	7.1E+02	8.5E+01	1.0E+04	1.0E+04	2.1E+03	2.4E+03	2.1E+03	1.2E+02	1.9E+03	1.4E+03	3.3E+04	1.2E+03
1.6E+03	1.1E+03	1.2E+02	1.6E+04	1.6E+04	3.3E+03	3.7E+03	3.2E+03	9.2E+02	3.0E+03	2.2E+03	5.1E+04	7.9E+02
1.0E+03	1.5E+03	2.0E+03	2.6E+04	2.5E+04	6.5E+03	7.8E+03	5.3E+03	1.7E+03	4.3E+03	2.1E+03	8.0E+04	3.8E+03
6.3E+02	9.8E+02	1.9E+03	3.9E+04	3.6E+04	4.0E+03	1.3E+04	1.8E+04	3.4E+03	2.7E+02	5.5E+03	1.3E+05	7.1E+03
4.0E+02	2.0E+04	8.1E+03	4.7E+04	8.1E+04	2.0E+04	1.5E+04	1.5E+04	1.0E+04	3.3E+03	8.4E+03	2.1E+05	5.1E+03
2.5E+02	6.3E+04	2.0E+05	5.1E+04	1.2E+05	1.3E+05	5.6E+04	1.2E+05	8.1E+04	4.1E+04	7.2E+04	3.7E+05	3.7E+04
1.6E+02	4.4E+04	3.3E+05	9.2E+04	1.2E+05	1.4E+04	5.1E+04	3.3E+04	1.3E+03	1.7E+04	3.3E+04	4.9E+05	3.3E+04
1.0E+02	1.6E+05	6.1E+05	1.3E+05	1.0E+05	3.0E+05	9.9E+04	1.2E+05	2.4E+04	0.0E+00	8.3E+04	9.4E+05	6.8E+05
6.3E+01	3.6E+05	1.1E+06	1.6E+06	1.8E+06	7.0E+04	5.4E+05	7.7E+05	1.6E+06	1.7E+04	8.3E+05	1.6E+06	1.3E+06
4.0E+01	8.7E+05	5.7E+05	6.2E+05	1.1E+06	9.2E+05	9.5E+05	1.7E+06	2.0E+06	3.3E+04	5.3E+05	1.7E+06	1.2E+06
2.5E+01	2.4E+06	3.8E+06	1.7E+06	7.0E+05	1.6E+06	1.9E+06	4.5E+05	8.2E+05	4.5E+05	5.0E+05	3.5E+06	1.2E+05
1.6E+01	2.6E+06	7.1E+06	2.3E+06	2.9E+06	6.1E+05	4.4E+05	1.0E+06	2.9E+06	6.7E+05	4.2E+06	7.4E+06	1.5E+06
1.0E+01	1.1E+05	1.2E+07	1.0E+06	3.1E+06	3.7E+06	7.9E+05	5.0E+05	1.9E+06	1.8E+06	3.3E+05	9.9E+06	6.7E+05
6.3E+00	8.8E+05	2.3E+07	1.0E+06	4.5E+06	1.1E+07	7.1E+05	1.8E+06	2.6E+06	1.3E+06	2.3E+06	1.1E+07	3.2E+06
4.0E+00	1.2E+06	4.1E+07	4.6E+06	3.7E+06	2.0E+07	5.8E+06	2.2E+06	3.9E+06	2.2E+06	3.5E+06	1.6E+07	1.7E+05
2.5E+00	5.3E+05	6.5E+07	1.7E+07	1.8E+07	3.2E+07	4.8E+06	1.7E+06	2.3E+07	2.1E+07	1.1E+07	2.9E+07	5.3E+06
1.6E+00	2.7E+05	7.9E+07	1.5E+07	8.6E+07	7.1E+07	2.0E+07	0.0E+00	2.1E+07	2.7E+07	5.2E+07	4.8E+07	5.0E+06
1.0E+00	1.4E+08	1.2E+08	2.8E+06	1.2E+07	1.1E+08	3.2E+07	6.7E+06	2.5E+07	7.2E+07	4.0E+07	8.2E+07	6.7E+06

Table S16. Standard deviation of the impedance measurements of α-pinene obtained with the TiO_2 (100% O_2)-thin-film-based sensor at different frequencies.

**Log10 Frequency (Hz)**	**Standard deviation of impedance (Ω)**											
	**0 ppm**	**68 ppm**	**109 ppm**	**205 ppm**	**315 ppm**	**410 ppm**	**506 ppm**	**602 ppm**	**712 ppm**	**807 ppm**	**903 ppm**	**999 ppm**
1.0E+06	3.7E+00	1.7E+00	3.5E+01	9.7E+00	3.5E+01	2.8E+01	3.0E+01	3.1E+01	4.0E+00	2.9E+01	3.6E+01	5.3E+01
6.3E+05	3.7E+00	2.5E+00	5.1E+01	7.5E+00	5.1E+01	4.8E+01	4.8E+01	4.8E+01	6.4E+00	4.8E+01	5.4E+01	9.3E+01
4.0E+05	4.9E+00	6.3E+00	8.0E+01	9.4E+00	8.4E+01	7.8E+01	7.8E+01	7.6E+01	1.1E+01	7.6E+01	8.4E+01	1.5E+02
2.5E+05	7.9E+00	1.2E+01	1.3E+02	1.5E+01	1.3E+02	1.3E+02	1.3E+02	1.2E+02	1.7E+01	1.1E+02	1.3E+02	2.5E+02
1.6E+05	1.2E+01	1.9E+01	2.1E+02	2.5E+01	2.1E+02	2.0E+02	2.0E+02	2.0E+02	2.9E+01	1.6E+02	2.1E+02	4.0E+02
1.0E+05	2.4E+01	3.3E+01	3.3E+02	4.3E+01	3.4E+02	3.3E+02	3.3E+02	3.2E+02	4.9E+01	1.9E+02	3.4E+02	6.4E+02
6.3E+04	3.6E+01	5.2E+01	5.2E+02	6.6E+01	5.3E+02	5.2E+02	5.2E+02	5.0E+02	8.1E+01	2.2E+02	5.4E+02	1.0E+03
4.0E+04	5.8E+01	8.3E+01	8.0E+02	1.1E+02	8.5E+02	8.3E+02	8.3E+02	8.0E+02	1.3E+02	2.4E+02	8.6E+02	1.6E+03
2.5E+04	8.9E+01	1.4E+02	1.2E+03	1.7E+02	1.3E+03	1.3E+03	1.3E+03	1.3E+03	2.0E+02	2.9E+02	1.3E+03	2.6E+03
1.6E+04	1.4E+02	2.1E+02	1.6E+03	2.8E+02	2.1E+03	2.1E+03	2.1E+03	2.0E+03	3.3E+02	3.9E+02	2.1E+03	4.0E+03
1.0E+04	2.4E+02	3.7E+02	1.8E+03	4.4E+02	3.4E+03	3.3E+03	3.3E+03	3.2E+03	5.2E+02	5.7E+02	3.4E+03	6.4E+03
6.3E+03	4.5E+02	5.5E+02	1.9E+03	6.7E+02	5.3E+03	5.2E+03	5.1E+03	5.0E+03	8.4E+02	8.8E+02	5.3E+03	1.0E+04
4.0E+03	3.1E+03	7.9E+02	2.2E+03	1.1E+03	8.5E+03	8.3E+03	8.2E+03	8.0E+03	1.2E+03	1.5E+03	8.4E+03	1.6E+04
2.5E+03	2.5E+03	1.3E+03	2.4E+03	1.6E+03	1.3E+04	1.3E+04	1.3E+04	1.2E+04	2.0E+03	2.0E+03	1.3E+04	2.5E+04
1.6E+03	2.1E+03	2.1E+03	3.3E+03	2.6E+03	1.9E+04	2.0E+04	2.0E+04	1.9E+04	3.3E+03	3.3E+03	2.1E+04	3.9E+04
1.0E+03	2.8E+03	2.9E+03	5.2E+03	4.2E+03	2.9E+04	3.1E+04	3.2E+04	3.0E+04	5.9E+03	6.7E+03	3.2E+04	6.0E+04
6.3E+02	1.0E+04	5.3E+03	2.8E+03	2.2E+03	2.9E+04	5.0E+04	4.7E+04	4.3E+04	6.9E+03	1.1E+04	5.2E+04	7.9E+04
4.0E+02	7.0E+03	2.1E+04	1.3E+04	4.5E+03	4.8E+04	5.5E+04	6.8E+04	7.3E+04	2.6E+04	8.0E+03	8.0E+04	1.3E+05
2.5E+02	8.0E+03	5.3E+04	7.9E+04	3.6E+04	1.1E+05	1.4E+05	5.7E+04	2.4E+04	1.3E+04	6.7E+04	1.0E+05	1.6E+05
1.6E+02	8.7E+02	2.4E+04	1.6E+04	5.6E+04	2.1E+04	1.6E+05	2.0E+05	2.2E+05	7.3E+03	2.7E+04	1.4E+05	1.9E+05
1.0E+02	1.9E+05	8.2E+04	3.6E+05	3.2E+05	3.1E+05	8.0E+05	3.3E+05	1.0E+06	4.4E+05	4.6E+05	5.8E+05	8.9E+05
6.3E+01	1.3E+06	1.2E+05	1.1E+06	6.1E+05	2.7E+06	1.2E+06	1.7E+06	6.8E+05	3.5E+05	1.3E+06	1.7E+06	3.2E+05
4.0E+01	1.4E+06	2.0E+06	6.8E+05	2.7E+06	2.9E+05	1.7E+06	1.7E+06	1.1E+06	3.6E+06	2.2E+06	4.4E+05	1.1E+06
2.5E+01	1.9E+06	7.0E+05	9.5E+05	1.2E+06	2.7E+03	9.0E+05	9.8E+05	5.0E+05	2.1E+06	9.1E+05	2.7E+06	1.1E+06
1.6E+01	3.8E+06	5.2E+05	4.9E+05	6.2E+06	3.2E+06	3.0E+06	6.3E+05	4.6E+06	5.2E+05	6.4E+05	3.8E+06	3.8E+06
1.0E+01	9.7E+05	1.8E+05	3.3E+05	4.0E+05	1.5E+05	1.4E+06	1.6E+06	1.1E+06	3.2E+05	4.7E+05	4.7E+05	2.0E+06
6.3E+00	2.9E+06	9.8E+05	6.2E+06	1.0E+06	2.0E+06	5.5E+05	6.8E+05	5.2E+06	3.8E+06	3.5E+06	2.4E+05	9.7E+05
4.0E+00	1.3E+07	3.3E+06	2.2E+06	3.6E+06	2.0E+06	3.2E+06	2.9E+06	6.2E+05	2.5E+06	1.5E+06	4.5E+06	7.0E+06
2.5E+00	5.8E+07	1.1E+07	4.6E+06	2.8E+05	4.8E+07	1.9E+05	1.2E+07	1.6E+07	3.7E+06	1.7E+06	3.2E+05	1.6E+08
1.6E+00	7.6E+07	5.0E+06	2.3E+07	1.2E+06	6.7E+04	6.7E+06	1.2E+07	3.7E+06	1.0E+06	1.0E+07	3.4E+06	8.3E+07
1.0E+00	1.4E+08	2.2E+08	1.5E+08	8.3E+07	4.9E+06	3.8E+06	1.5E+07	4.4E+07	6.8E+06	1.4E+08	3.7E+07	1.3E+08
